# Use of Recovered Carbon Black from Waste Tires in Triple Mesoscopic Stack Perovskite Solar Cells

**DOI:** 10.1021/acssusresmgt.4c00422

**Published:** 2025-02-03

**Authors:** Susana Iglesias-Porras, Amy Neild, Lee Stevens, Wei Li, Colin Snape, Owen Woodford, Niall Straughan, Elizabeth A. Gibson

**Affiliations:** † Energy Materials Laboratory, School of Natural and Environmental Science, Newcastle University, Newcastle Upon Tyne NE1 7RU, UK; ‡ Energy Technologies Building, University of Nottingham - Jubilee Campus, Wollaton Road, Nottingham NG8 1BB, UK

**Keywords:** recovered carbon black, circular economy, perovskite solar cells, outdoor testing, net-zero targets, PV waste, semiconductor supply risks, energy resilience

## Abstract

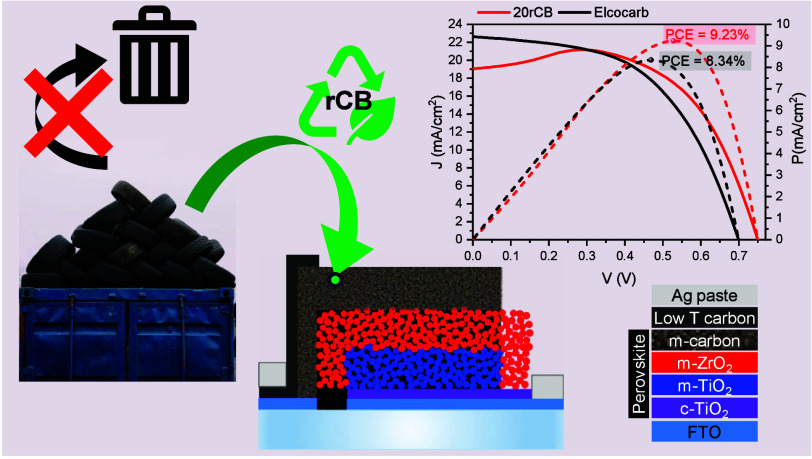

This research addresses critical challenges in the photovoltaic (PV) industry to achieve net-zero greenhouse gas emissions by 2050, amidst geopolitical semiconductor supply risks and escalating volumes of PV waste. We demonstrate the opportunity to address these challenges through the design of PV cells which are compatible with a circular economy. In this proof-of-concept study, unpurified locally sourced recovered carbon black (rCB) from waste tires was integrated into the mesoporous carbon layer of triple mesoscopic perovskite solar cells as a sustainable alternative to virgin carbon sources, and comparable efficiencies (9.98%) to commercial carbon paste benchmarks (10.4%) were attained. Key findings reveal that the presence of sulfur, silica, and zinc oxide contaminants only affected performance and durability marginally. While sulfur enhanced perovskite crystallization, as evidenced by an increased fill factor, it potentially influenced the absorber’s valence band maximum, slightly dropping the open-circuit voltage. Silica and zinc oxide exacerbated moisture ingress under UK weather conditions, as revealed by outdoor testing, which accelerated degradation post-breaching of the encapsulant. Such degradation could be mitigated through effective encapsulation. Although further research is crucial to maximize performance and device longevity, the feasibility of using locally sourced rCB in PV technology has been demonstrated. This approach could support regional energy resilience and sustainability objectives within a circular economy framework.

## Abstract 2

This research explores integrating recycled carbon black from waste tires into solar cells to improve sustainability in the photovoltaic industry.

## Introduction

Achieving net zero greenhouse gas emissions by 2050 is crucial to limit global warming to 1.5 °C above pre-industrial levels while meeting the energy demands of a growing global population. Current PV technology has the potential to deliver over 25 times the global power target set for 2050 with net-zero emissions,^1^ yet two critical concerns threaten this achievement: potential geopolitical disruptions in semiconductor supply chains and the increasing volume of PV waste. China dominates the Silicon PV industry,^2^ hosting over 70% of metallurgical-grade silicon production, polysilicon production, and silicon PV module manufacturing.^3^ This concentration of the market increases the risk of disruptions to supply, potentially impacting global PV deployment and threatening sustainable energy resilience in many countries. By 2050, meanwhile, PV waste generation is expected to exceed the initial projection of 78 million tonnes,^4^ as global cumulative PV capacity in 2023 increased by more than double that predicted in 2016.^5^ In the UK, plans to reach 70 GW of installed solar capacity by 2035 could double or triple their predicted PV waste volumes by 2065.^6^ While recycling the silicon back into PV is technically feasible and could mitigate supply chain risks, the current mainstream industrial recycling strategy fails to recover silicon or produce metallurgical-grade material, focusing on metal recovery and downcycling high-value materials into lower-grade products.^7^

Addressing these challenges requires reframing the linear model of PV product consumption into a circular model. The circular economy is a system where products and materials are kept in circulation at their highest value by ensuring recyclability by design. This allows nature the ability to regenerate by extracting fewer raw materials and eliminating the accumulation of waste in landfill. Third generation photovoltaics are presented in the scientific literature as an excellent opportunity to fit into this circular model of consumption. Halide perovskites are recyclable ionic semiconductors, which are synthesized using earth abundant materials at low temperatures,^8,9^ with record power conversion efficiencies matching those of silicon.^10^ Although using precious metals in the counter electrode of top performing devices makes them more susceptible to supply limitations and increased costs, there are alternative architectures.^11^ In the triple mesoscopic stack (TMS), metal electrodes are substituted with carbon inks, which reduces the costs, facilitates scale-up, increases the availability of the components and extends the device longevity.^11^ These carbon inks are manufactured as a combination of graphite and carbon black and are commonly fabricated from virgin sources.

Carbon black (CB) can be recovered from waste tires at a rate of 0.30 to 0.40 kilograms per kilogram of tire processed.^12^ Approximately 1.5 billion tires reach their end of life yearly,^13^ which, assuming an average tire weight of 9 kg, transforms into 13.5 million metric tonnes of end-of-life material and potentially up to 5.4 million metric tonnes of recovered carbon black (rCB) per year. This rCB typically sells at a lower price compared to virgin CB and has a market valued at USD 1.88 billion in 2022, with predictions to reach USD 5.42 billion by 2033.^14^ There is significant potential in using this valuable resource from waste streams in high-value applications such as in electronic devices or as electrodes for power generation and storage.

In this proof-of-concept study, we substituted the virgin carbon black in the carbon electrode of TMS perovskite solar cells with recovered carbon black from waste tires provided by Wastefront, a rubber waste recycling company who are establishing a large-scale facility in the Northeast of England. Our results prove the feasibility of using unpurified and locally sourced rCB in perovskite solar cells which attain equivalent efficiencies to those made with commercially available carbon paste, under local weather conditions. This demonstrates the opportunity provided by the circular economy in supporting regional energy resilience and sustainability goals.

## Experimental Section

### Chemicals and Materials

For the fabrication of the lab made carbon paste, zirconium(IV) oxide (nanopowder, <100 nm particle size), ethyl cellulose (48.0–49.5% (w/w) ethoxyl basis) and graphite (powder, <20 μm, synthetic) were purchased from Sigma-Aldrich. The α-terpineol (97+%) solvent and the virgin carbon black (CB, Carbon black, acetylene, 100% compressed, 99.9+%) were acquired from Thermo Scientific. The recovered carbon black was provided by Wastefront, as a byproduct of their tire pyrolysis process described in their filed patent applications,^15,16^ and used as received.

For the perovskite solution preparation Gamma Butyrolactone (GBL, Thermo Scientific Chemicals, 99+) and methanol (MeOH, Fisher Chemical, Extra Dry, for Synthesis) were used. The organic cation salt methylammonium iodide (MAI) and the additive 5-ammonium valeric acid iodide (AVAI) were purchased from Greatcell solar. The lead precursor PbI_2_ (99.99%) was acquired from TCI chemicals. For device fabrication, carbon (Elcocarb B/SP) and zirconium oxide pastes (Zr-Nanoxide ZT/SP) were purchased from Solaronix. The mesoporous titanium dioxide paste (Titania paste, transparent) was acquired from Sigma-Aldrich. For the deposition of the spray coated TiO_2_ layer, acetylacetone (≥99.5%) and titanium(IV) isopropoxide were purchased from Sigma-Aldrich. Absolute ethanol (99.8%) was acquired from Thermo Scientific.

All chemicals were used without further purification.

### Carbon Paste Fabrication

Four grams of total carbon (sum of graphite and carbon black) were introduced into a ball mill mixing jar with 0.4 g of ethyl cellulose, 60 mg of ZrO_2_ and 8 mL of terpineol and ball milled for 2 h at 300 rpm with seven 1 cm balls. The ratio of carbon black to graphite was changed from 100:0, 80:20, 50:50, 30:70, 20:80 and 10:80 to fabricate the 100%, 50%, 30%, 20% and 10% virgin (CB) and recovered (rCB) carbon pastes.

### Perovskite-Carbon Film Fabrication for Photophysical Characterization

The mesoporous carbon layers were applied to glass substrates by a doctor blade and annealed at 400 °C for 30 min. Subsequently, they were infiltrated with the MAPbI_3_-5-AVAI precursor solution via doctor blade and annealed at 50 °C for 5 h, similar to the full device. A reference MAPbI_3_-5AVAI perovskite film was slot-die coated directly onto the glass substrate under the same deposition and annealing conditions. For the PL and TRPL measurements, the samples were illuminated from the carbon side.

### Solar Cell Fabrication

Fluorine-doped tin oxide (FTO, Pilkington, 7 Ω sq^–1^) coated glass substrates were prepared by brushing with a 2% Hellmanex in water solution followed by rinsing with de-ionized water, acetone, and ethanol. Each rinse was dried with a nitrogen flow and ozone cleaned for 15 min before subsequent layer depositions. A compact TiO_2_ film was deposited using spray pyrolysis (15 cycles) on a hot plate at 450 °C with 3.6 mL of acetylacetone and 2.4 mL of titanium(IV) isopropoxide dissolved in 54 mL of ethanol. Afterwards, a mesoporous TiO_2_ film was screen printed using a polyester 165.31 mesh and sintered at 450 °C for 30 min. Substrates were laser etched (ULYXE DPSS laser marker) to separate contacts, followed by screen printing and sintering of mesoporous ZrO_2_ at 500 °C. Then, a mesoporous carbon layer was applied by a doctor blade, and the complete stack was annealed at 400 °C. All sintering steps included a 30 min ramp-up, a 30 min annealing stage and natural cooling to room temperature. The mesoporous layer thicknesses averaged 1.53 μm for m-TiO_2_, 2.32 μm for m-ZrO_2_, and 15–20 μm for carbon, as shown in Figure S1 of the Supporting Information.

The perovskite solution was prepared by following the protocol from Hashmi *et al*.^17^ Modifications to this recipe included substituting 10% of the GBL solvent with methanol, where noted. Before stack-infiltration, the edges of the samples were coated with petroleum jelly to prevent the perovskite from coating the metal contact area. The perovskite solution was then infiltrated into the TMS using slot-die coating (Ossila) with a recipe of 30 μL solution per sample at a speed of 12 mm s^–1^. Annealing was then conducted for 5 h in air at 50 °C. Subsequently, a humidity treatment was performed in an enclosed atmosphere at 40 °C for 12 h using a saturated binary salt solution (NaCl) to maintain around 75% relative humidity (RH).^18^ The protective petroleum jelly was removed with acetone-soaked cotton buds and low-temperature carbon was deposited on the positive contact and dried at 100 °C for 10 min. Finally, silver paint was applied to both positive and negative contacts.

Samples were encapsulated before outdoor deployment using a two-part epoxy resin (Araldite, RS UK) around the edges of the carbon with a glass slide on top. Pressure was applied with clips until the resin reached its full strength after 2 h.

### Characterization

The X-ray diffraction (XRD) spectra were conducted on a Bruker D2 PHASER Cu X-ray diffractometer in a Bragg Brentano setup, where the k_β_ radiation was removed with a 0.2 Ni filter and the Cuα_2_ stripped from the Cuα_1_ (λ = 1.5418 Å) using the Rachinger method in the X’pert Highscore Plus software. The XPS survey, C 1s and valence scans were acquired with a Thermo Scientific K-Alpha X-ray Photoelectron Spectrometer (XPS). BET adsorption/desorption isotherms were acquired in powder samples with a Micromeritics ASAP 2420 instrument. Approximately 0.5 g of powdered samples was placed into a sample tube and degassed at 150 °C for 15 h under high vacuum (<0.013 mbar) to remove moisture and other adsorbed gases. Pore volume and size distribution were calculated by combining CO_2_ isotherms at 0 °C (0.004–1.180 bar) and N_2_ isotherms (0.00001–0.99500 relative pressure) at −197 °C using the NLDFT model based on carbon slit pores. The ultra micropore volume (0–0.7 nm), micropore volume (0–2 nm), mesopore volume (2–50 nm) and total pore volume (0–100 nm) were calculated using the cumulative pore volume generated from this model.

The BET equation is
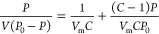
where *P* = partial pressure of the adsorbate gas; *P*_0_ = saturation pressure of the adsorbate gas; *V* = volume of adsorbed gas; *V*_m_ = monolayer adsorbed gas volume; *C* = BET constant (the C parameter).

Each carbon paste Kr isotherms (at −197 °C, 0.12–0.65 relative pressure) generated were the result of the average of 3 individual mesoporous carbon samples after subtraction of the weight of the empty tube and FTO glass substrates. All were degassed prior to analysis for 15 h at 120 °C under high vacuum (<0.013 mbar). Surface area, micropore volume and limited mesopore volume (from 2–20 nm) were calculated using the Derjaguin–Broekhoff–deBoer (DBdB) model.^19^ The SEM/EDX images were obtained by using a JSM IT510 Scanning Electron Microscope. Steady state photoluminescence (PL) and time-resolved photoluminescence (TRPL) measurements were carried out in an EPL 635 Edinburgh Instruments with a laser excitation at a wavelength of 635 nm (30 mW at 10 MHz), 200 kHz (30 mJ s^–1^) and spot size 0.0625 cm^2^ at the sample. Sheet resistance measurements were acquired with an Ossila Four-Point Probe, thicknesses were measured with a Bruker DektakXT profilometer, and microscopy images were acquired with a Celestron 44340 LCD digital microscope. Thermogravimetric analysis (TGA) data was obtained with a Perkin Elmer STA 6000 and contact angle measurements were taken with a KSV CAM100.

The current density–voltage (JV) characteristics of fabricated devices were measured using an Ossila Source Meter under Air Mass 1.5 Global (AM 1.5 G) with a Wavelabs solar simulator intensity of 1 sun (100 mW cm^–2^). JV scans were also taken at lower light intensities between 0.1 and 1 sun for reference against outdoor data (Supporting Information Figure S23). The IPCE spectra were conducted by using a Bentham PVE300. For outdoor characterization an in-house built system was used to measure JV scans every hour during daylight hours (Ossila Source Meter Unit). Environmental data was acquired simultaneously with the JV scans, including temperature at the surface of the cell (ATC Semitec 103JT-025 Jt Thermistor), ambient temperature and humidity (DHT22 Digital Temperature and Humidity Sensor Module), and light intensity (OSRAM BPW21 Photodiode, calibrated against AM 1.5 G spectrum between 0.01 and 1 suns using the Thorlabs NDK01 Neutral Density filter kit).

## Results and Discussion

### Chemical and Structural Characterization

Two carbon paste series were fabricated with increasing amounts of graphite following the methodology reported in the Experimental Section using virgin carbon black (CB) and recovered carbon black from waste tires (rCB) as provided by Wastefront. Commercial carbon black paste (Elcocarb) was used as a reference given its common use for the fabrication of lab-made TMS devices,^20,21^ and directly compared to the 20% CB and 20% rCB mixtures containing the optimum carbon black to graphite weight ratio according to the literature.^22^

**Figure 1:**
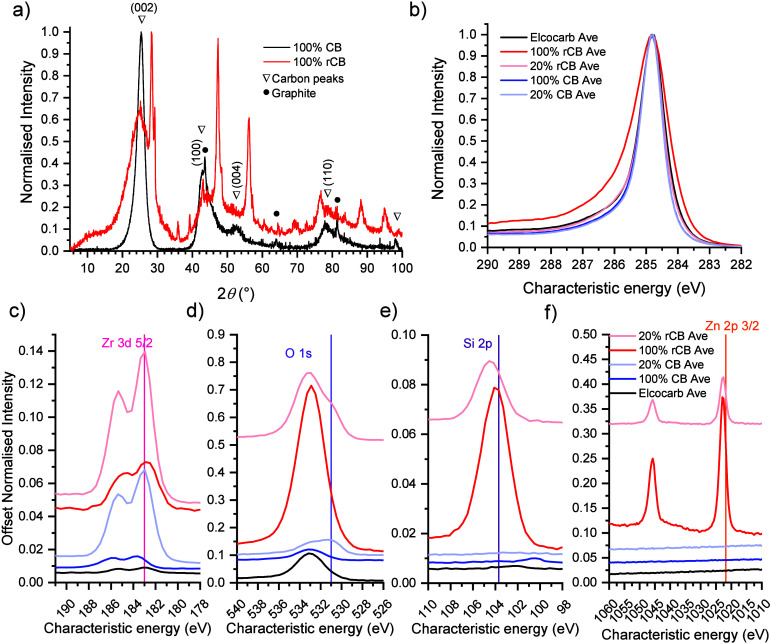
(a) Normalized XRD pattern of 100% CB powder and 100% rCB powder. (b) Normalized XPS C 1s peak from mesoporous film samples made with Elcocarb, 100% CB paste, 100% rCB paste, 20% CB paste, and 20% rCB paste. Normalized Zr 3d 5/2 (c), O 1s (d), Si 2p (e), and Zn 2p 3/2 (f) peaks of mesoporous film samples of Elcocarb, 100% CB, 100% rCB, 20% CB, and 20% rCB pastes. All peaks are normalized to the carbon C 1s.

The first significant difference between the pastes was found in the carbon structure, as shown in Figure 1. CB exhibited higher crystallinity, and a more graphitic nature compared to its recovered counterpart, evident from the sharpness of the carbon peaks in the powder XRD diffraction pattern (Figure 1a) and the normalized C 1s peak in mesoporous film samples detected by XPS (Figure 1b). Adding more graphite to the CB pastes (Supporting Information Figure S2a) barely changed the shape of the C 1s peak. In contrast, the rCB series showed the opposite trend (Supporting Information Figure S2b), with the C 1s line in the 20% rCB film shifting to a position similar to the Elcocarb reference. The broader C 1s peak in the rCB samples is often attributed to carbon atoms in small aromatic compounds that attach during the carbon black recovery process from waste tires.^23^

**Figure 2:**
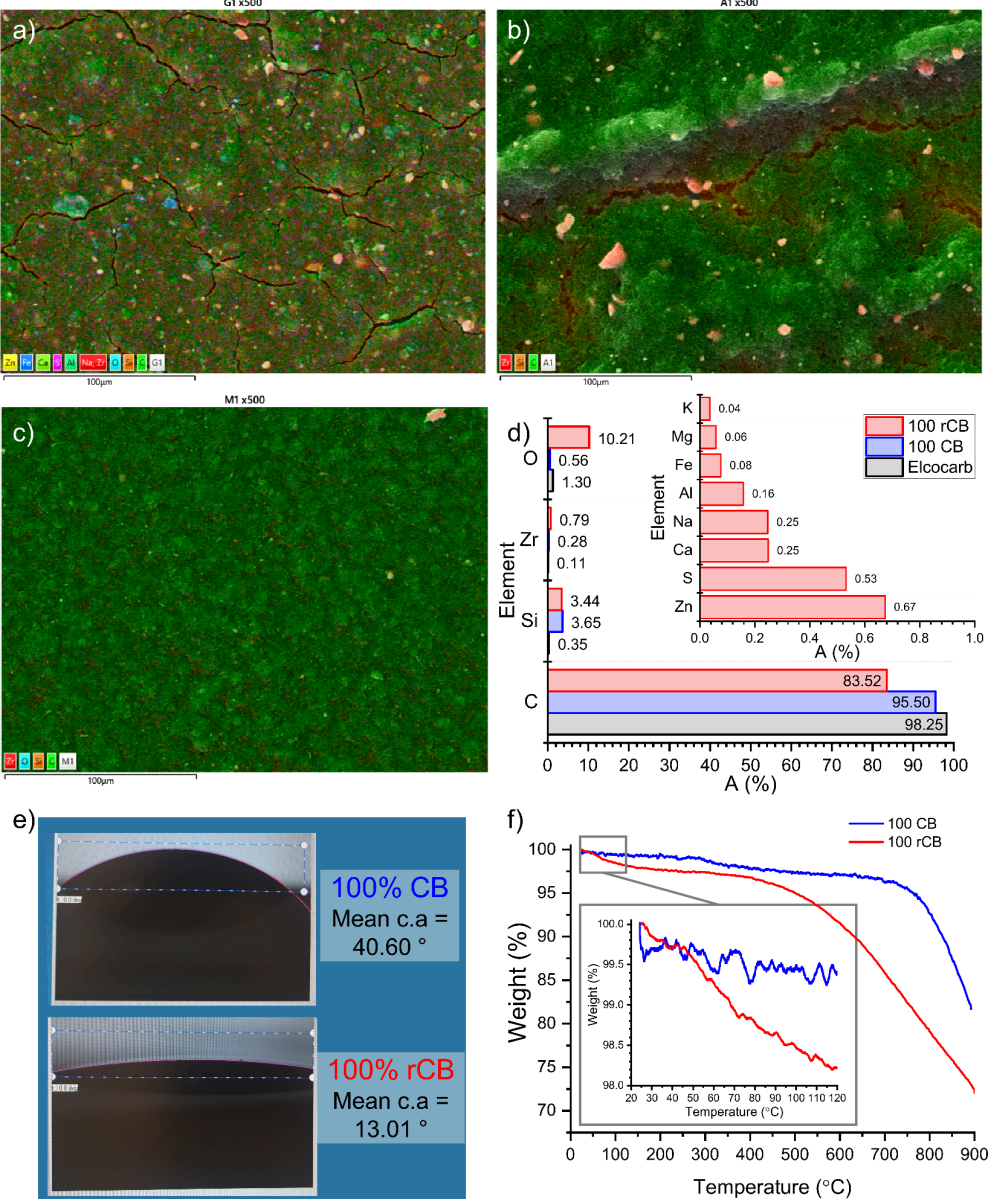
SEM/EDX top view of mesoporous film samples made with 100% rCB (a), 100% CB (b), and Elcocarb (c). A plot with the percentage of main elemental components detected by EDX can be seen in part d. (e) Contact angle (c.a.) measurement of 100% CB (top) and 100% rCB (bottom) against a droplet of water. (f) TGA scans for 100% CB and 100% rCB powder samples with a detailed inset between 20 °C and 120 °C, showcasing the higher proportion of water in the rCB sample.

The presence of the main inorganic components of the paste (C and ZrO_2_) was confirmed by the XPS survey scan in all CB, rCB and Elcocarb samples (Supporting Information Figure S3a), which were calibrated relative to the reference C 1s peak position (284.8 eV). The XPS analysis showed all film samples presented surface contamination with F, as determined by the F 1s signal at 690 eV (Supporting Information Figure S3b), with no traces of fluorine appearing in the EDX images (Figure 2).^24^

The maximum of the Zr 3d 5/2 peak was at 182.9 eV in most samples, which is consistent with the characteristic feature of tetragonal ZrO_2_ (Figure 1c, Supporting Information Figure S4a and Figure S4b).^25,26^ Similarly, spectra with the highest proportion of ZrO_2_ also contained a clear shoulder at ca. 530.6 eV in the O 1s peak, which is in agreement with the reported O–Zr binding energy.^25,26^ Although this component is present in all samples (Figure 1d, Supporting Information Figure S4c and Figure S4d), the dominant O 1s peak at 533 eV in the 100% CB, 100% rCB and Elcocarb samples was consistent with the presence of water molecules adsorbed onto the surface.^27−29^ In the case of the rCB series, the intensity of this peak was enhanced significantly, partly due to the presence of SiO_2_, with the Si 2p signal at 103.7 eV (Figure 1e). Additionally, a larger quantity of surface adsorbed water appeared to be present in the rCB compared to CB. This was indicated by contact angle measurements (Figure 2e) and TGA (Figure 2f). Greater hydrophilicity was displayed by the 100 rCB sample compared to the 100% CB by its lower contact angle (Figure 2e). A higher mass loss below and around 100 °C in the TGA scans performed under N_2_ (Figure 2f), indicated a greater proportion of water in the rCB compared to CB. Increased water adsorption in the rCB could be explained by the hydrophilic inorganic impurities such as silica, zinc oxide, alumina, and calcium carbonate.^30−32^ Another potential factor influencing higher adsorption of water could be a larger presence of functional groups in the carbon such as −OH, −NH_2_, −COOH, or −SO_3_H.^33^

Interestingly, the Zr 3d 5/2 peak intensity increased with increasing content of graphite for both the CB and rCB series (Figure 1e, Supporting Information Figure S4a and Figure S4b). This was despite all recipes having the same ratio of ZrO_2_ to carbon, and the trend extracted from the EDX data is consistent with this (Supporting Information Table S1 and Table S2). This apparent accumulation of this metal oxide on the surface when the graphite content in the paste was raised above 30% is due to inhomogeneity in the sample arising from the large size of the graphite flakes present in the first iteration of paste (Supporting Information Figure S6), inhibiting the homogeneous distribution of the ZrO_2_. We also noted a much higher content of ZrO_2_ in the lab fabricated pastes compared to the commercial Elcocarb both through XPS (Figure 1e) and EDX (Figure 2), which we reduced in subsequent synthesis of new batches of paste along with a reduction of the graphite flake size.

The contaminants S, Ca, and Na were uniformly distributed in the recovered carbon paste samples, as shown in EDX maps (Figure 2, Supporting Information Figure S7) and XPS valence scans (Supporting Information Figure S5b). Trace elements such as Fe, Al, Mg, and K were detected by EDX (Figure 2d, Supporting Information Figure S7). The presence of Si in EDX maps originated primarily from the substrate, as indicated by its concentration along the sample cracks (Supporting Information Figure S7). However, Si was also distributed across the sample since silica is a common filler present in the ash of recovered carbon black from pyrolyzed waste tires.^23,34^

As anticipated, the atomic proportion of contaminants significantly decreased as the graphite content of the rCB pastes was increased (Supporting Information, Table S1 and Table S2). In the state-of-the-art 20% rCB composition, these elements constituted less than 0.37 at. %. TGA performed under air revealed an inorganic ash content of 20 wt % in the 100% rCB carbon powder (Supporting Information Figure S11), corresponding to 4 wt % for the 20% rCB paste.

**Figure 3:**
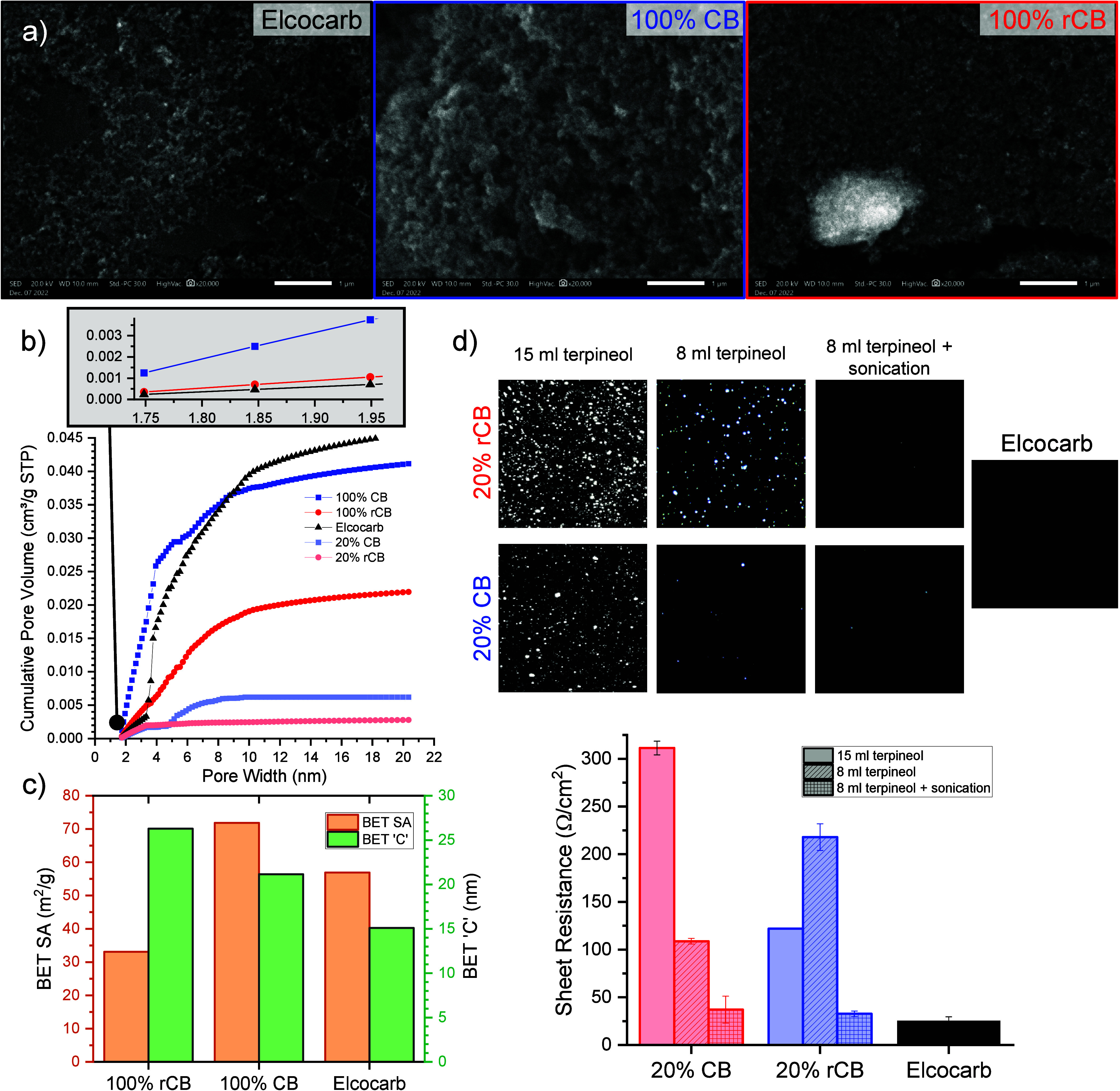
(a) SEM showing the morphology of films made with 100% rCB, 100% CB, and Elcocarb pastes. (b) Cumulative pore volume. (c) Surface area and ‘C’ parameter extracted from BET measurements of 100% rCB, 100% CB, and Elcocarb films. (d) Average sheet resistance of 20% CB, 20% rCB, and Elcocarb films, subject to different dilutions and/or treatments.

Morphologically, the most distinctive feature of the rCB paste was its lower pore volume compared to the lab-made CB paste and the commercial Elcocarb. This was apparent both in the SEM images and BET results (Figure 3a–c). The Kr adsorption/desorption isotherm data along with the calculated pore size distribution can be found in Figure S12 of the Supporting Information. These findings oppose the porosity values observed in the carbon black powders alone, where the cumulative pore volume of the rCB was significantly larger than that of the CB (Supporting Information Figure S13). These may be related to the preparation process, for example, the presence of impurities blocking pores.

Known pore altering factors like the amount of binder and solvent, mixing conditions, deposition, and annealing steps were kept constant for both CB and rCB pastes.^35−37^ Both the zirconium IV oxide and graphite have low surface areas, micro and mesopore volumes. The former 22.3 m^2^ g^–1^, 0.0125 cm^3^ g^–1^ and 0.1527 cm^3^ g^–1^ and the latter 8.76 m^2^ g^–1^, 0.0274 cm^3^ g^–1^ and 0.0312 cm^3^ g^–1^ respectively. As the zirconia was kept constant, the graphite was shown to have the greatest effect of reducing pore volume and surface area. The low pore volumes and surface area of graphite will dilute or reduce the surface area and pore volumes of the CB and rCB in the paste. The TGA revealed that the rCB mixture contained more water, volatile organic compounds and polycyclic aromatic hydrocarbons before annealing, as can be seen in the higher mass loss below and around 100 °C, 100–400 °C and 400–600 °C,^38^ respectively, in Figure 2f. These contaminants could affect the pore formation dynamics during sintering.^39,40^ This resulted in more compact regions separated by cracks (Supporting Information Figure S8). Additionally, contaminants like metal oxides, sulfides, and silica, could clog pores and create a more packed structure, reducing the surface area and pore volume.

The BET results for the pastes with higher graphite content showed a significant decrease in pore volume and surface area, which could be attributed to the graphite being of low porosity, reducing overall porosity, and the large graphite flake size blocking pores (Supporting Information Figures S6 and S12e and h). With further optimization of the paste, the flake size was reduced to match that of the literature optimum (∼10 μm),^11^ and the Elcocarb paste (Supporting Information Figure S9 and Figure S10).

Despite the lower porosity of the 100% rCB sample, its BET C parameter was the highest at 26.3, followed by the 100% CB paste at 21.15, and the Elcocarb sample at 15.1 (Figure 3c). The BET C parameter reflects the interaction strength between the adsorbate (Kr gas) and the adsorbent (carbon film), particularly the energy of adsorption in the first adsorbed layer or monolayer, indicating differences in adsorption energy and affinity due to factors such as chemical composition, surface functionalization, roughness, pore size distribution, or impurities.^33,41^ The high micropore content in the 100% CB paste explains its higher C value compared to Elcocarb, as micropores have stronger intermolecular forces of attraction (van der Waals forces) in pores <2 nm, adsorbing Krypton gas molecules more strongly. Alternatively, the 100% rCB paste’s highest C value could be due to the presence of more impurities and functional groups, changing the surface energy of the carbon film, increasing the strength of adsorption, and potentially explaining its higher water adsorption capacity.^33^

Rounds of paste optimization involved using various volumes of terpineol and sonication treatments. The microscope images of the mesoporous films in Figure 3d show that reducing the solvent volume from 15 mL to 8 mL per 4 g of carbon mix decreased pinhole formation, thereby reducing the sheet resistance of the lab-made CB paste. Interestingly, despite the changes in appearance, the conductivity of the rCB paste did not improve with thickening. A 6-hour sonication treatment was crucial to reduce the sheet resistance of both CB and rCB pastes to values comparable to the commercial Elcocarb paste. For virgin carbon black, an acceptable sheet resistance and fewer pinholes were achieved after 30 min of sonication, as shown in Figure S14 of the Supporting Information.

#### Photophysical Characterization

**Figure 4:**
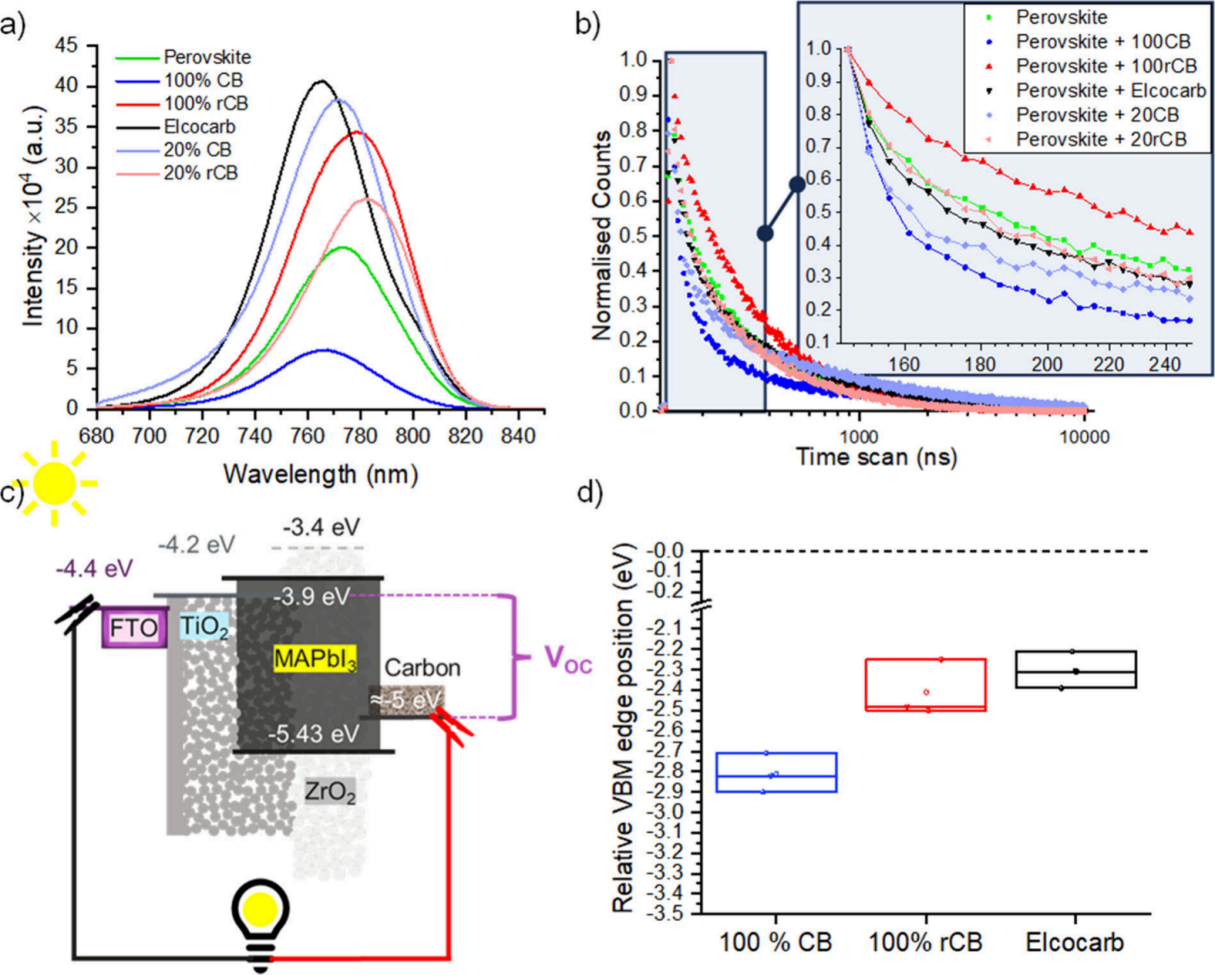
PL (a) and TR-PL (b) scans for carbon films made with Elcocarb, 100% rCB, 100% CB, 20% rCB, and 20% CB with a layer of MAPbI_3_ perovskite deposited on top. A film of MAPbI_3_ perovskite deposited on glass is used as a reference. (c) Energy band diagram of the triple mesoscopic perovskite cell. (d) Relative Valence Band Maximum (VBM) position of 100% CB, 100% rCB, and Elcocarb carbon films extracted from the XPS valence band edge fit.

Steady state (PL) and time-resolved photoluminescence (TRPL) decay measurements were performed to evaluate the charge transfer at the perovskite/carbon interface with the commercial, CB and rCB pastes as illustrated in Figure 4a and b, respectively. To do so, mesoporous films of 100% CB, 100% rCB, 20% CB, 20% rCB, and Elcocarb were fabricated and infiltrated with MAPbI_3_-5AVAI as reported in the Experimental Section. A bare MAPbI_3_-5AVAI perovskite film was deposited onto the glass as a reference using the same methodology. The photophysical characterization was done exclusively on such films, to establish direct comparisons between the different carbons and the perovskite film.

The normalized TRPL decay data was analyzed using a bi-exponential decay model, and the parameters are detailed in Table S4 of the Supporting Information. To support the interpretation of the TRPL, we also measured the relative position of the VBM of the mesoporous carbon films using XPS. Charge injection dynamics between the perovskite and the charge transport layers depend on the position of the electronic bands. Carbon has a VBM around −5.0 eV, creating a significant gap with the valence band of MAPbI_3_ at −5.43 eV (Figure 4c).^42,43^ Changes in the composition of the carbon layer can affect the depth of the valence band edge position, and therefore affect charge extraction and open circuit voltage values. Figure 4d displays a boxplot of the relative position of VBM for the pure carbon black pastes (100% CB and 100% rCB) against the commercial paste, performing 3 measurements per sample. The position was obtained from fitting an edge down function to the XPS valence scans between 6.5 and 0 eV as presented in Figure S5a of the Supporting Information.

The results in Figure 4 evidence a highly heterogeneous system, making it challenging to extract definitive trends. Several processes occur which determine the PL intensity and rate of decay, including carrier trapping due to intrinsic defects, surfaces, grain boundaries and impurities, bimolecular quenching, and charge extraction. The porous scaffold adds further complexity. However, several noteworthy observations warrant discussion.

Firstly, quantum confinement is evident from the blue shifts in the photoluminescence of the Elcocarb, 100CB, and 20CB samples compared to the bare perovskite. Specifically, the blue shifts for the CB and Elcocarb samples correlate with predominant pore sizes of 3.8 and 3.9 nm, respectively (Figure S12). This finding is consistent with prior studies linking reduced perovskite particle size, especially in metal oxide templates, to noticeable blue shifts in PL spectra.^44,45^ In the Elcocarb sample, quantum confinement seems to dominate, resulting in higher PL intensity, potentially due to enhanced quantum yield. In 20CB samples, quantum confinement may also play a role, indicated by their higher PL intensity relative to 100CB.

Lower PL intensity was observed for the 100CB sample compared to Elcocarb and 20CB. 100CB exhibits improved band alignment, as shown by XPS, enhancing charge extraction. This is further supported by the faster quenching observed in TRPL for the 100CB samples. The reduced amount of carbon black in the 20CB likely accounts for its slower TRPL decay. The deepening of the valence band in 100CB samples may result from a higher proportion of bonds with passivating groups like hydrogen, hydroxyl, carbonyl, or carboxyl groups.^46^ CB has a larger surface area and higher reactivity than Elcocarb (Figure 3c), increasing the number of surface atoms and dangling bonds,^47,48^ which alter the VBM.^49,50^

Another critical factor is the role of dopants in the rCB samples. Sulfur and chalcogenides have been shown to enhance perovskite crystal growth and passivate defects. Sulfur atoms can effectively coordinate with Pb^2+^ at the interface between perovskite and electron transport layers (ETLs) like SnO_2_ or TiO_2_, passivating surface and interface defects through their strong electron-donating ability.^51,52^ This effect also applies to sulfur-containing molecules,^53−55^ which are effective precursors for improving crystal growth in TMS solar cells.^56^ The passivating effects of sulfur could explain the red shift in steady PL observed in the 100 and 20 rCB samples, dominating over potential quantum confinement shifts. Although the TRPL decay is slower in the 100 rCB samples, the valence band position was found to be similar to Elcocarb, indicating that contaminants in the XPS valence spectra did not affect the valence band position significantly. However, sulfur-containing impurities may affect the interface between the perovskite and the carbon, possibly deepen the VBM of the perovskite, and position it lower in energy than carbon and creating a small barrier to extraction. This barrier would account for the slower TRPL decay and higher steady PL intensity in these samples.

In the 20 rCB samples, the effects of impurities appear more balanced compared to the 100% case. This balance results in improved crystal quality relative to the bare perovskite, as evidenced by its higher PL intensity and slightly faster extraction capabilities, as observed in the TRPL measurements, which indicate a reduced misalignment of the valence bands.

#### Solar Cell Assembly and Characterization

**Figure 5:**
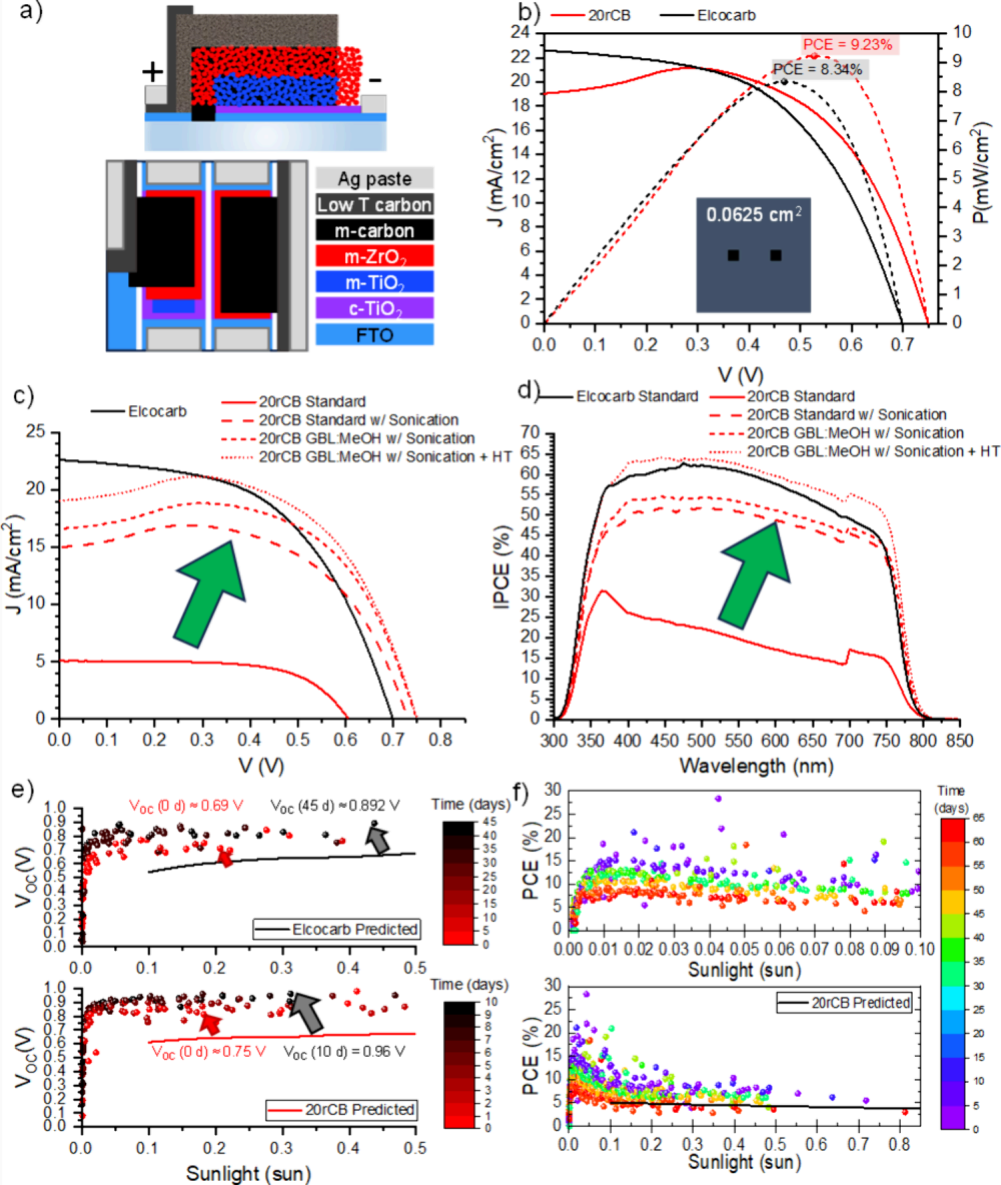
(a) Structure of the TMS devices. (b) JV and Power–Voltage curves for the top performing Elcocarb and 20% rCB samples, measured with a small area of 0.0625 cm^2^. JV scans (c) and IPCE (d) of top performing devices from several batches showcasing changes in the performance and infiltration of the perovskite as treatments like sonication of the rCB paste, change in the solvent mixture of the perovskite solution (GBL:MeOH), and exposure to a hydration treatment (HT). (e) Improvement in *V*_OC_ over time (colormap) as a function of light intensity for the 20% rCB and Elcocarb samples under outdoor conditions, compared to measured values in the lab. (f) Evolution of PCE over time (colormap) under outdoor conditions of the 20% rCB sample as a function of light intensity compared to the measured values in the lab.

TMS solar cells were slot-die coated following the protocol defined in the Experimental Section, after optimising the coating speed and volume of solution using the Elcocarb reference paste (Supporting Information Figure S16 and Figure S17). Note that limitations to the average and maximum device performances are discussed in Section 8 of the Supporting Information. To further improve the performance, a layer of “low temperature” carbon ink was deposited between the edge of the mesoscopic carbon and the edge of the device, after the perovskite annealing and before the deposition of silver paste (Figure 5a). This approach doubled the average performance of standard devices, as shown in Figure S15 of the Supporting Information.

For the fabrication of the rCB devices, the state-of-the-art 20-to-80 carbon black-to-graphite ratio was used (20% rCB) and the device performance was compared against the reference Elcocarb paste. This time, the graphite size was optimized to enable an adequate level of porosity to suitably infiltrate the stacks with perovskite solution. By tailoring the perovskite solution to the carbon layer porosity and enhancing the connectivity of the rCB paste, similar performances were achieved between the top 20% rCB and Elcocarb solar cells, as evidenced by the JV scan of top performing devices of 20% rCB and Elcocarb (Figure 5b).

The infiltration of the perovskite was improved through several batch iterations: (a) applying a 6-hour sonication treatment to the 20% rCB paste which limited the formation of aggregates in the carbon layer (Supporting Information Figure S19), (b) reducing the density of the MAPbI_3_ solution with 10% methanol to further enhance percolation through the stack (Supporting Information Figure S20), and (c) performing a 12-hour post-annealing hydration treatment at 40 °C and 75% relative humidity to induce water-assisted recrystallization (Supporting Information Figure S21).^17,57^ These changes resulted the progressive increase of both the best cell’s *J*_SC_ values from 4.95 mA cm^–2^ up to 19.06 mA cm^–2^ and *V*_OC_ from 0.587 V up to 0.745 V (Figure 5c). Improvements in the infiltration were also reflected in the IPCE of the 20%rCB devices, doubling from 32% up to 64% (Figure 5d). The optimum rCB device achieved a maximum power conversion efficiency (PCE) of 9.23%, surpassing the reference device and closely matching the Elcocarb top sample with a PCE of 8.34%.

Although the open circuit voltage of the top-performing rCB device measured with a small area mask surpassed that of the Elcocarb, the overall trend showed a slight decrease in *V*_OC_ for rCB compared to the commercial reference, but better compared to the less optimal CB devices (Supporting Information Figures S19d, S20d, and S21d). It is possible that contaminants such as sulfur/sulfides could enhance the crystallization but also lower the VBM of the perovskite.^55,58^ However, any sulfur-enhanced crystallization or charge-transfer evidently allowed the rCB devices to exhibit an increased fill factor (FF) compared to the Elcocarb reference in all batches (Supporting Information Figures S19e, S20e, and S21e).

Accounting for the drop in *V*_OC_ due to large shading of the active area could increase the efficiency to 9.98% and 10.4%, respectively (Table 1, see the Supporting Information for a more detailed discussion). The Elcocarb top-performing sample’s IPCE could also improve with a hydration treatment applied to a properly infiltrated sample, according previously studies on TMS devices.^17,57^ However, in this work, no clear performance improvement was observed for the Elcocarb samples fabricated with the GBL:MeOH solution, due to poor infiltration (Supporting Information Figures S20 and S21). Further optimization of the deposition speed and volume for the 20 rCB devices using the GBL:MeOH solution is likely to further enhance the efficiency, considering batch-to-batch variations (Supporting Information Figure S23).

**Table 1:** Solar Cell Parameters Extracted from JV Curves of Top Performing rCB and Elcocarb Samples Measured with a 0.0625 cm^2^ Mask^a^

Sample	*J*_SC_ (mA/cm^2^) @ 0.0625 cm^2^	*V*_OC_ (V) @ 0.0625 cm^2^	FF @ 0.0625 cm^2^	PCE (%) @ 0.0625 cm^2^	*V*_OC_ (V) @ 0.64 cm^2^	PCE_est_ (%) @ true 0.0625 cm^2^
Elcocarb	22.6	0.694	0.532	8.35	0.861	10.4
20 rCB	19.1	0.745	0.650	9.24	0.806	9.98

a The potential performance PCE_est_ was estimated using the values of *V*_OC_ measured with a masking area close to the active area of the device (0.64 cm^2^).

#### Outdoor Testing of Solar Cells

To evaluate the durability of the devices under real outdoor conditions, Elcocarb, CB, and rCB samples were tested outdoors in North Yorkshire (UK) for 63 days (March 27^th^ to May 29^th^, 2024). The weather conditions during this period, shown in the Supporting Information Figure S24a, featured predominantly low light and high humidity. Over 67% of daylight measurements were below 0.1 sun, and more than 50% registered over 70% relative humidity (RH). Despite ventilation, the ambient temperature of the measuring box often exceeded 25 °C, peaking at 40.2 °C due to sunlight-induced overheating of the solar cells’ surface. Temperature fluctuations between day and night, often exceeding 30 °C, posed a challenge for the durability of the encapsulation.

Before outdoor deployment, the samples were sealed with epoxy resin (Araldite), which proved unsuitable for the high surface temperatures of the cells, recording up to 90.51 °C as shown in Figure S24b and S24c of the Supporting Information. The adhesive bond weakened at 80 °C, allowing moisture to penetrate within days. These high temperatures also accelerated device degradation due to the high volatility of the MA compound.^59−61^

The performance improvement of the carbon devices within 24 h of encapsulation and storage in the desiccator, right before field deployment, was attributed to water-aided recrystallization and slow solvent evaporation.^17,57,62^ This improvement is shown by the gap between predicted and observed cell parameters in Figures 5e and 5f and Figure S25 in the Supporting Information. Similar enhancements were observed for earlier batches, with average performance increasing by up to 0.6% after 4 days in the desiccator (Supporting Information Figure S22f).

The speed of cell performance improvement and degradation was proportional to the moisture volume on the carbon layer as shown in Figure 5e. The *V*_OC_ of the rCB cell improved faster than for the Elcocarb reference, reaching its peak in 10 days versus 45 days for Elcocarb. Both samples were made and encapsulated identically, so solvent evaporation and water-induced recrystallization should have occurred at similar times. However, the hygroscopic impurities in the rCB apparently attracted more water, accelerating both performance improvement and decay. This was combined with temperatures in the measuring box that mimicked the HT treatment conditions. The increase of moisture volume on the rCB samples in combination with light and temperature could have also triggered further decomposition processes given the presence of sulfide contaminants in the carbon, such as the formation of sulfuric acid, PbS or other reactive species.

The performance decay over time against light intensity for the rCB cell is shown in Figure 5f. The cell maintained relatively stable power for 10–15 days but showed significant degradation after 30 days, reaching T_80_ relative to its maximum power. After one month, rCB samples displayed more yellow PbI_2_ spots than the CB or Elcocarb (Supporting Information Figure S24d). By 60 days, the performance had dropped below the predicted T_80_ value due to a critical loss of *J*_SC_ and *V*_OC_, performing worse than after manufacturing (Supporting Information Figure S25).

A similar decay trend was observed in the Elcocarb and CB devices, although over a longer timeframe, which we attribute to the lower hygroscopicity of the carbon layer (Supporting Information Figures S24 and S25). After 3 weeks in a desiccator post-outdoor exposure, JV scans at 1 sun confirmed full degradation, with all devices showing less than 10% of their original PCE within three months.

## Conclusion

We have demonstrated that integrating recovered carbon black into triple mesoscopic perovskite solar cells can lead to comparable efficiency (9.98%) to commercial benchmarks (10.4%), despite the noticeable presence of contaminants such as sulfur, zinc and silica. Despite the presence of these contaminants, the rCB devices had a higher FF than the commercial benchmark. On average, a slight decrease in the *V*_OC_, and *J*_SC_ compared to the benchmark were observed, due to a combination of electronic and textural differences. These were shown to affect the infiltration and crystallization of the perovskite absorber, and charge-extraction. For the best-performing devices, however, a higher *V*_OC_ was attained, which we attribute to the deepening of the VBM for rCB compared to the virgin material. Furthermore, after an initial rapid improvement in performance, the hygroscopic nature of the contaminants in rCB exacerbated moisture ingress under outdoor exposure, accelerating cell degradation following encapsulation breaches. This issue can be mitigated with effective encapsulation and pre-encapsulation drying. Our findings highlight the potential of utilizing unpurified recovered carbon black from waste tires, supporting a circular economy of the PV industry by valorizing waste materials without requiring prior purification. Further optimization is necessary to enhance performance and device longevity. It is possible that the pyrolysis conditions and further purification could be optimized to produce a carbon material with more tailored properties for solar cell applications. This proof-of-concept study underscores the promise of sustainable materials in advancing photovoltaic technology while contributing to environmental stewardship through resource conservation and waste reduction efforts.

## Supporting Information Available

Mesoporous layers thicknesses, XPS scans, SEM and EDX maps, BET measurements, optical microscopy images, sheet resistance measurements, fit values for TRPL, JV parameters and IPCE of several batches of solar cells, calibration JV scans of solar cells at various light intensities before outdoor deployment, outdoor temperature measurements on the surface of the cells, and outdoor JV parameters of solar cells as a function of time and light intensity (PDF)

## Abbreviations

AM: air mass
AVAI: ammonium valeric acid
BET: Brunauer–Emmett–Teller
CB: virgin carbon black
EDX: energy dispersive X-ray spectroscopy
ETL: electron transporting layer
FF: fill factor
FTO: fluorine-doped tin oxide
GBL: gamma butyrolactone
HOMO: highest occupied molecular orbital
HT: hydration treatment
HTM: hole transporting material
IPCE: incident photon to current conversion efficiency
JV: current density-voltage
*J*_SC_: short-circuit current density
MAI: methylammonium iodide
MeOH: methanol
PCE: power conversion efficiency
PL: photoluminescence
PTFE: polytetrafluoroethylene
PV: photovoltaic
rCB: recovered carbon black
RH: relative humidity
SEM: scanning electron microscopy
TGA: thermogravimetric analysis
TMS: triple mesoscopic stack
TRPL: time resolved photoluminescence
UV: ultraviolet
*V*_OC_: open-circuit voltage
VBM: valence band maximum
XPS: X-ray photoelectron spectroscopy
XRD: X-ray diffraction
