# Supplementary material for: Use of Recovered Carbon Black from Waste Tires in Triple Mesoscopic Stack Perovskite Solar Cells
Source: ACS Sustain Resour Manag. 2025 Feb 3;2(2):322–33. doi: 10.1021/acssusresmgt.4c00422 (PMC11874465; doi:10.1021/acssusresmgt.4c00422)
Supplement: Supplementary file 1 — rm4c00422_si_001.pdf [file rm4c00422_si_001.pdf]

Supporting Information

Use of Recovered Carbon black from Waste Tyres in Triple Mesoscopic Stack Perovskite Solar Cells

Susana Iglesias-Porras[†*], *Amy Neild*[†], *Lee Stevens*[††], *Wei Li*[††], *Colin Snape*[††], *Owen Woodford*[†], *Niall Straughan* [†], *Elizabeth A. Gibson*[†]*.

[†]Energy Materials Laboratory, School of Natural and Environmental Science, Newcastle University, Newcastle Upon Tyne, NE1 7RU, UK.

[††]Energy Technologies Building, Jubilee Campus, Wollaton Road, Nottingham, NG8 1BB, UK.

Contents

Section 1. Supplementary experimental methods information 2
Section 2. Supplementary XPS information 3
Section 3 Supplementary SEM and EDX information 6
Section 4. Supplementary TGA information 10
Section 5. Supplementary BET information 11
Section 6. Supplementary microscopy and sheet resistance information 13
Section 7. Supplementary TRPL information 15
Section 8. Supplementary device fabrication and optimisation information 16
Section 9. Supplementary Outdoor Test information 26
REFERENCES 29

Section 1. Supplementary experimental methods information

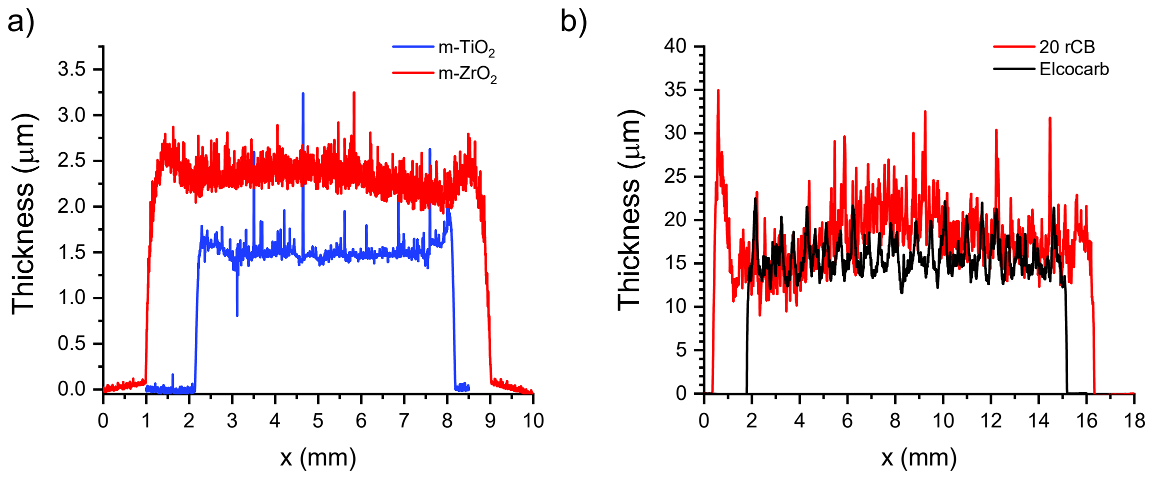

Figure S1. Thickness of the mesoporous TiO_2, ZrO_2 layers (a), and commercial versus recovered carbon (b), obtained via profilometry.

Section 2. Supplementary XPS information

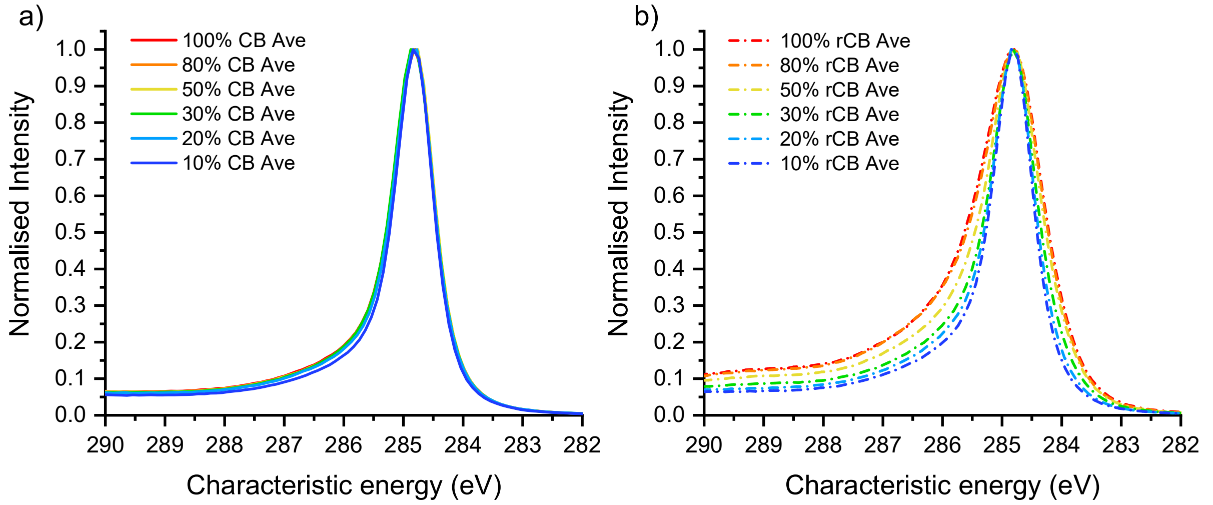

Figure S2. Normalised value of the average C 1s line in XPS obtained from a range of carbon black:graphite pastes using CB (a) and rCB (b).

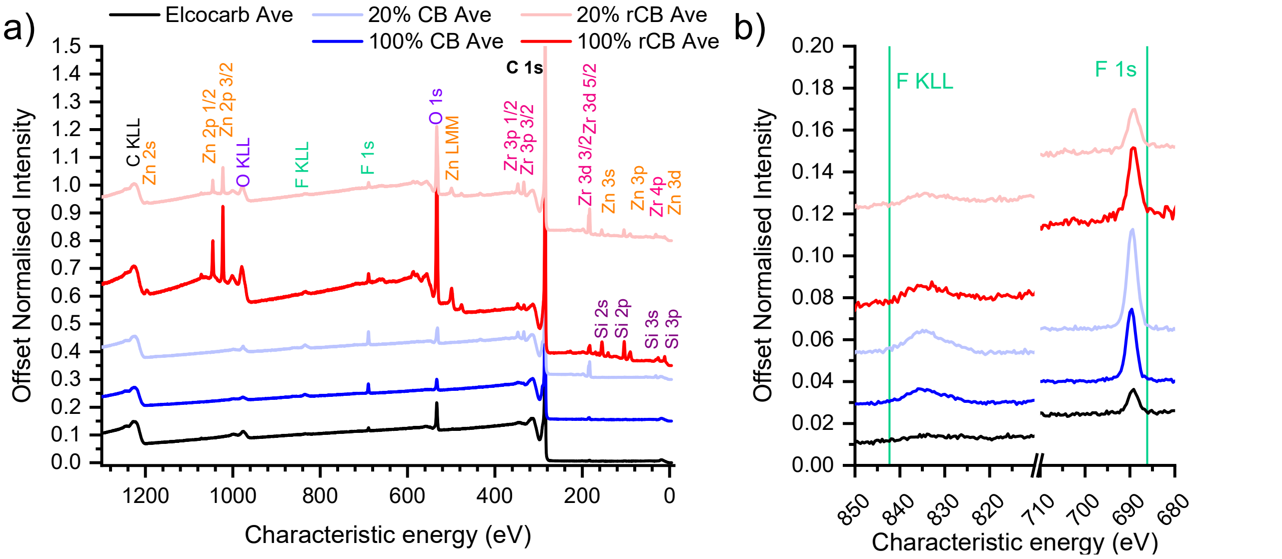

Figure S3. (a) Normalised XPS average survey scan for mesoporous film samples of Elcocarb, 100% CB, 100% rCB, 20% CB and 20% rCB pastes. (b) Detailed sections of the F 1s scan.

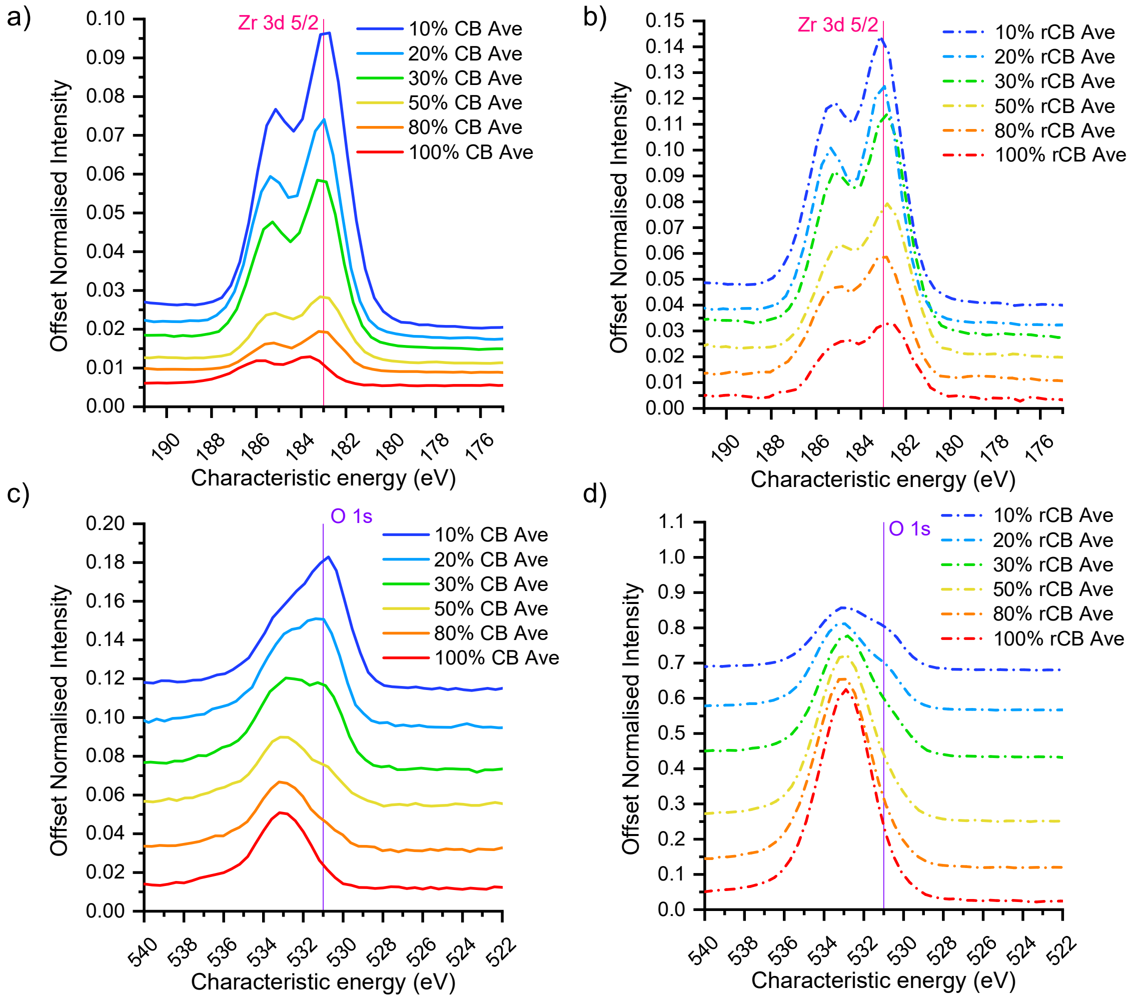

Figure S4. XPS Zr 3d and O 1s core level spectra for the CB (a, c) and rCB (b, d) film paste series.

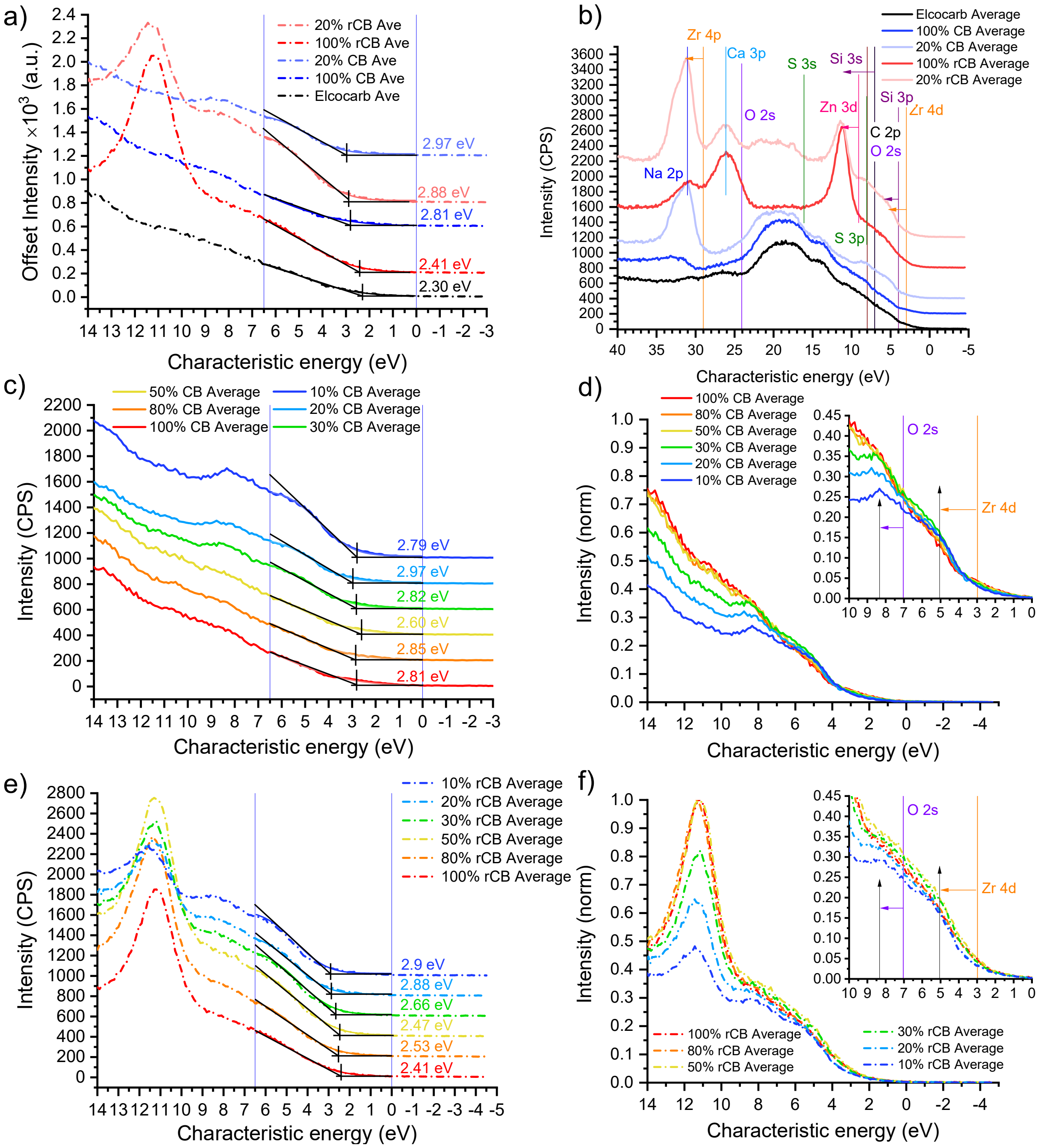

Figure S5. (a) Valence band XPS scan of 100% CB, 100% rCB, 20%CB, 20% rCB and Elcocarb samples fit with an edge down function. Identification of elements in the scan is included in (b). Valence band XPS scans of the CB paste series and rCB paste are included in (c) and (e). Detail on the effects of ZrO_2 on the valence edge of the normalised scan for each of the series is included in (d) and (f), respectively.

Section 3. Supplementary SEM and EDX information

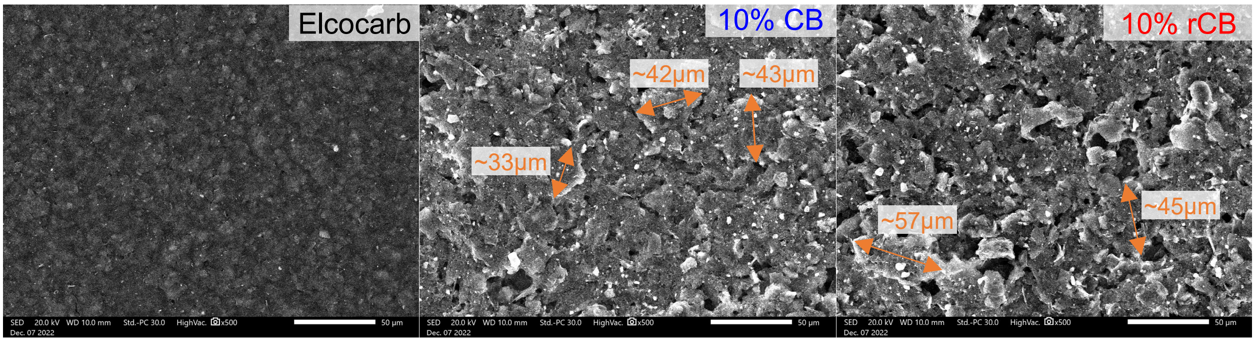

Figure S6. Comparative view of graphite flake size between Elcocarb (left), 10% CB (middle) and 10% rCB (right) film samples.

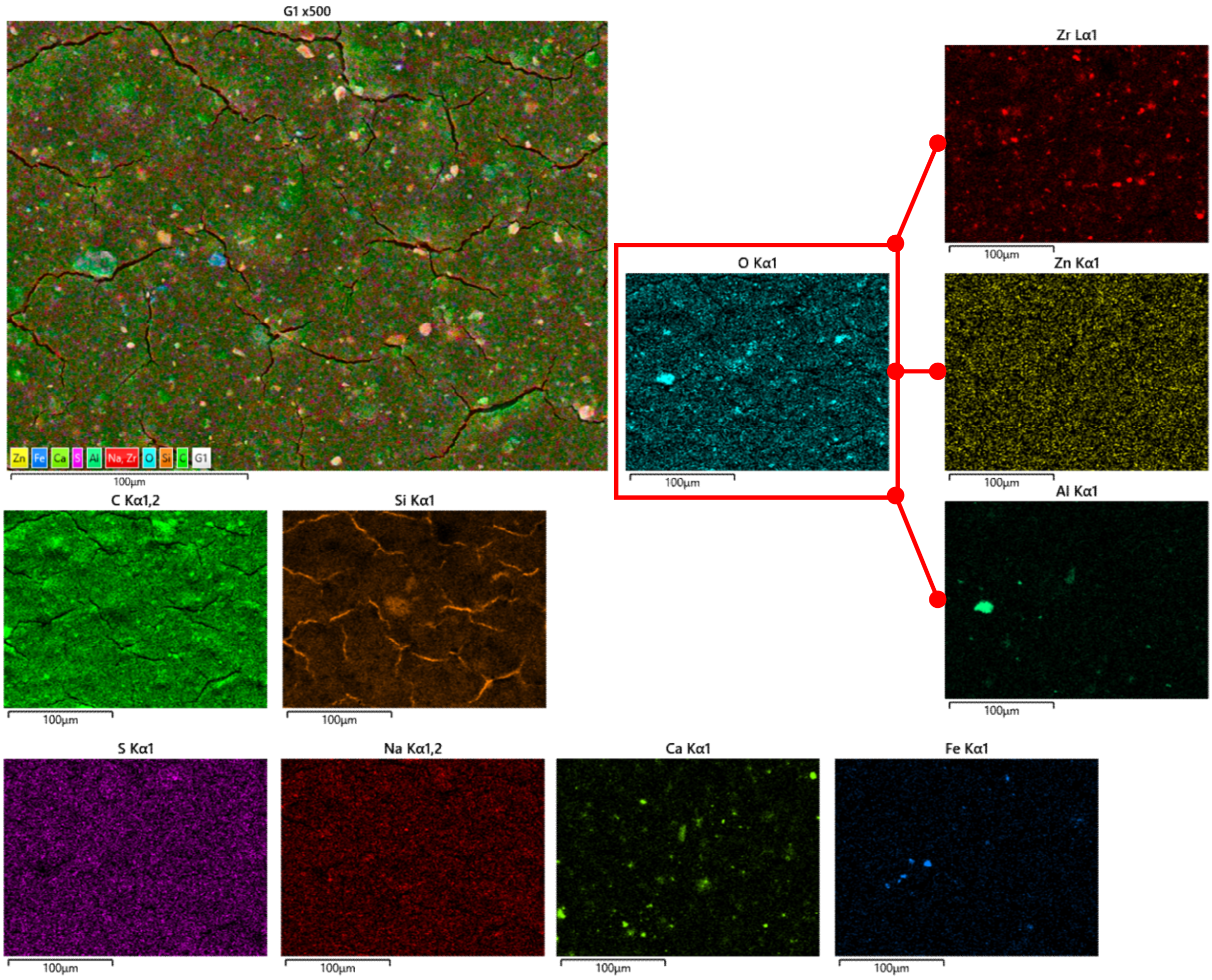

Figure S7. EDX map of 100% rCB paste broken down in elemental components.

	% element present in sample					
**% of CB in paste**	**100**	**80**	**50**	**30**	**20**	**10**
C	95.503	96.529	97.585	96.392	96.398	95.383
Si	3.655	2.276	0.587	0.779	0.184	1.386
Zr	0.281	0.476	0.653	0.832	0.919	0.811
O	0.561	0.719	1.109	1.930	2.418	2.351
F	0.000	0.000	0.067	0.068	0.068	0.068

Table S1. Atomic proportion of elements in CB carbon paste film samples.

	% element present in sample					
**% of CB in paste**	**100**	**80**	**50**	**30**	**20**	**10**
C	83.522	85.494	89.104	91.301	91.645	93.436
Si	3.440	3.059	2.069	2.132	2.275	1.589
Zr	0.794	0.942	0.770	0.759	0.802	0.835
O	10.213	8.980	7.263	5.323	4.908	3.938
F	0.000	0.000	0.000	0.000	0.000	0.069
Zn	0.674	0.496	0.289	0.163	0.122	0.060
S	0.532	0.395	0.211	0.125	0.083	0.041
Ca	0.248	0.176	0.101	0.066	0.033	0.033
Na	0.247	0.184	0.118	0.058	0.058	0.000
Al	0.158	0.104	0.050	0.049	0.049	0.000
Fe	0.076	0.076	0.024	0.024	0.024	0.000
Mg	0.058	0.058	0.000	0.000	0.000	0.000
K	0.036	0.036	0.000	0.000	0.000	0.000

Table S2. Atomic proportion of elements in rCB carbon paste film samples.

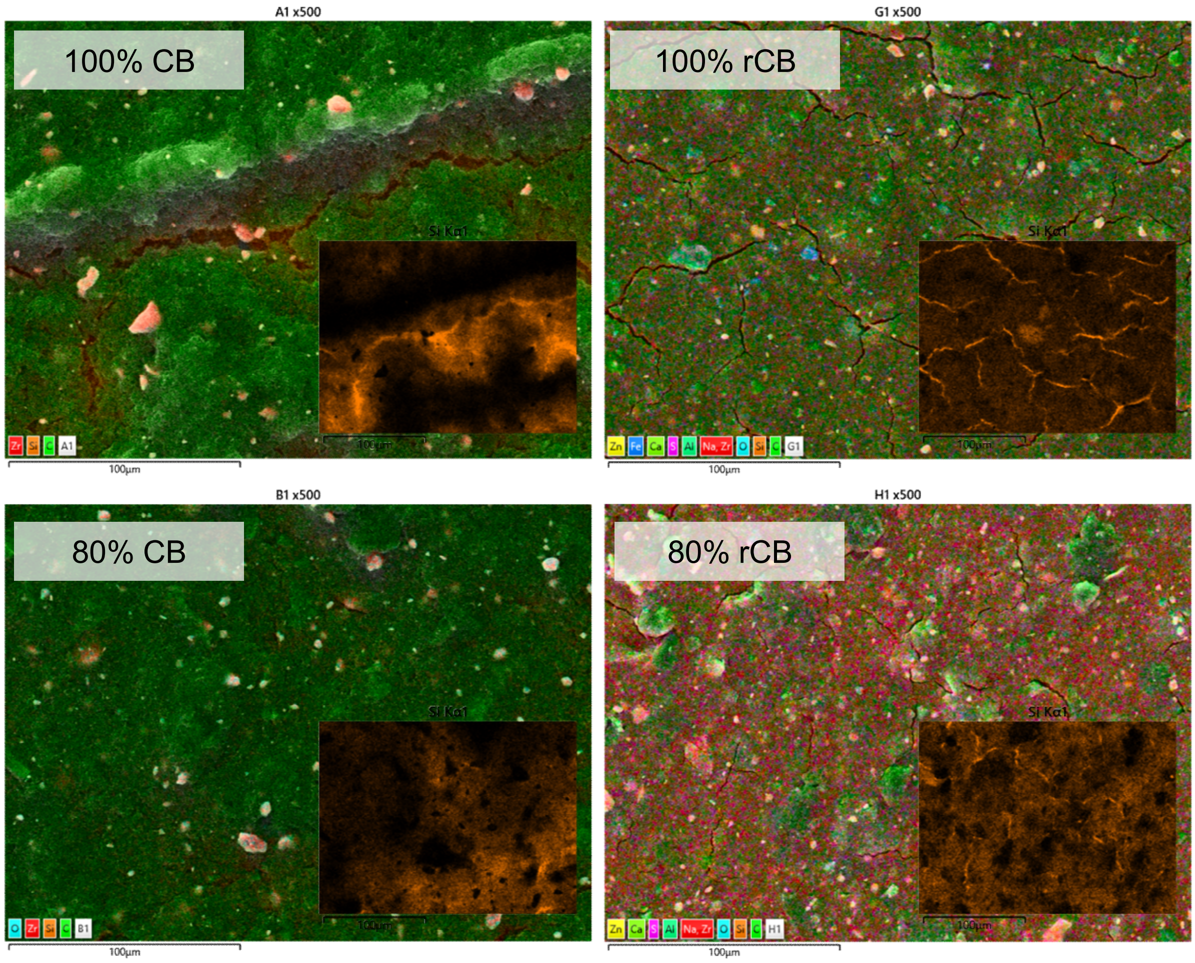

Figure S8. Difference in the presence of cracks on the surface of SEM/EDX scans for 100% CB and 80% CB compared to 100% rCB and 80% rCB. An EDX map of Si is included in the corner of the images highlighting the exposure of the silicon wafer substrate through the cracks.

Due to the large graphite flake size detected in the first iteration of paste formulation, the graphite flake size was reduced using ball milling. The optimum size was found after 3 hours of ball milling at 500 rpm as represented in Figure S8.

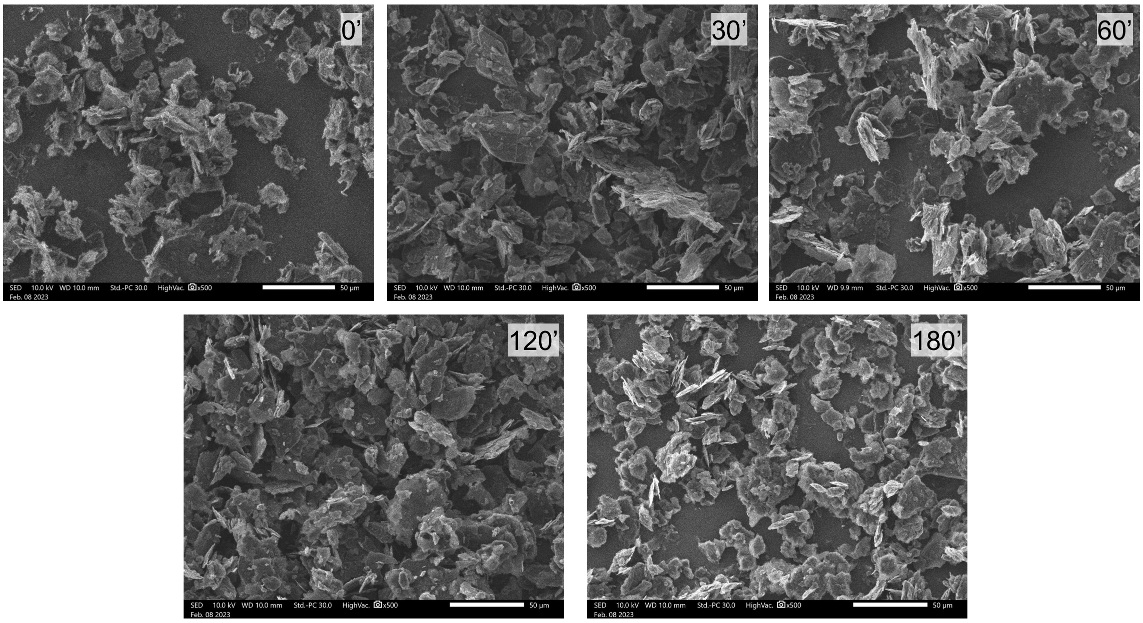

Figure S9. SEM images of graphite flakes ball milled for 0, 30, 60, 120 and 180 minutes at 500 rpm.

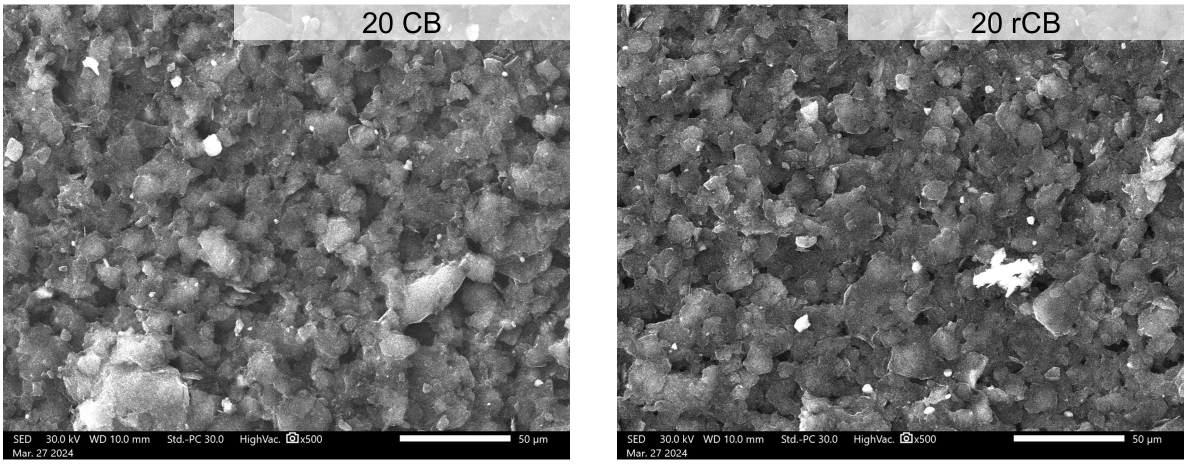

Figure S10. SEM image of 20CB (left) and 20 rCB (right) optimised pastes with reduced graphite flake size.

Section 4. Supplementary TGA information

Samples were scanned both under N_2 and air, following the same protocol: heating from 25 °C to 900 °C at 10 °C/min, keeping a 5-minute isotherm at 900 °C and then cooling down until 25 °C (also at 10 °C/min).

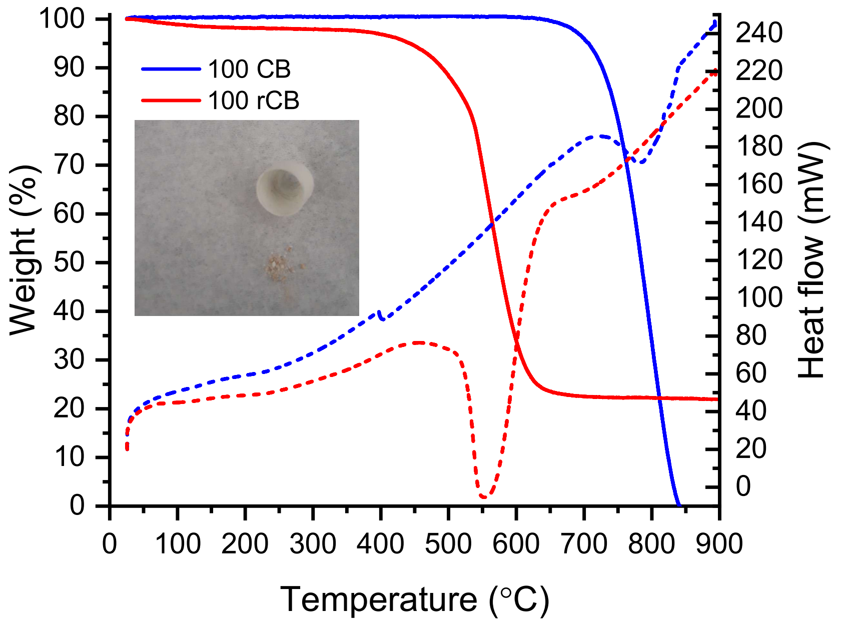

Figure S11. TGA and DSC scans for the 100% CB and 100% rCB carbon powder samples under air. The inset showcases an image of the residue left in the 100% rCB sample after completing the heating cycle.

Section 5. Supplementary BET information

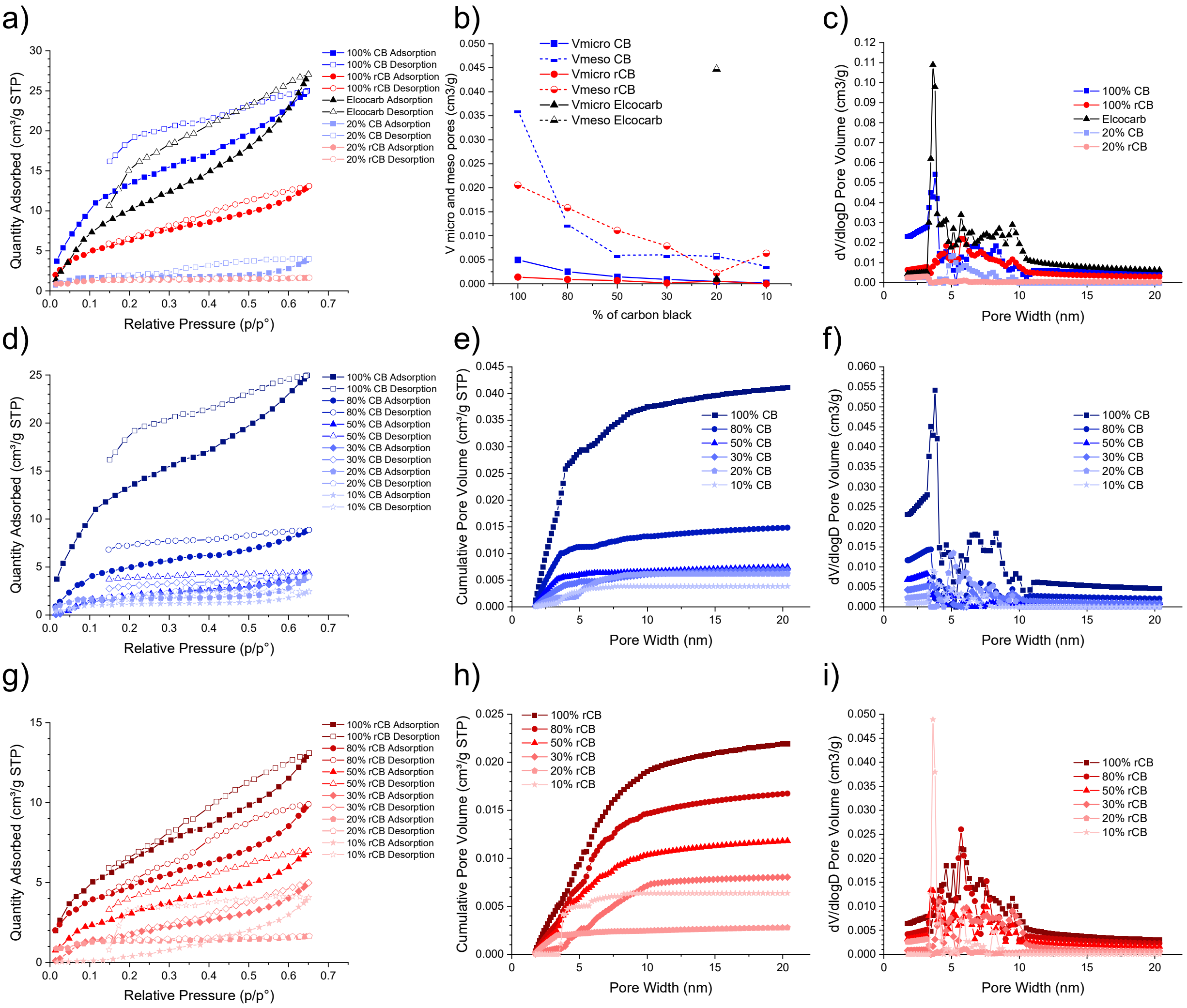

Figure S12. Kr adsorption/desorption curves for 100% CB, 100% rCB, 80% CB, 80% rCB and Elcocarb samples (a), CB sample series (d), and rCB sample series (g). b) Comparison between the volume of micro and mesopores in the 100% CB, 100% rCB, 80% CB, 80% rCB and Elcocarb samples. Cumulative pore volumes of the CB sample series (e), and rCB sample series (h). Pore volume density represented as a function of pore width for the 100% CB, 100% rCB, 80% CB, 80% rCB and Elcocarb samples (c), CB sample series (f), and rCB sample series (i).

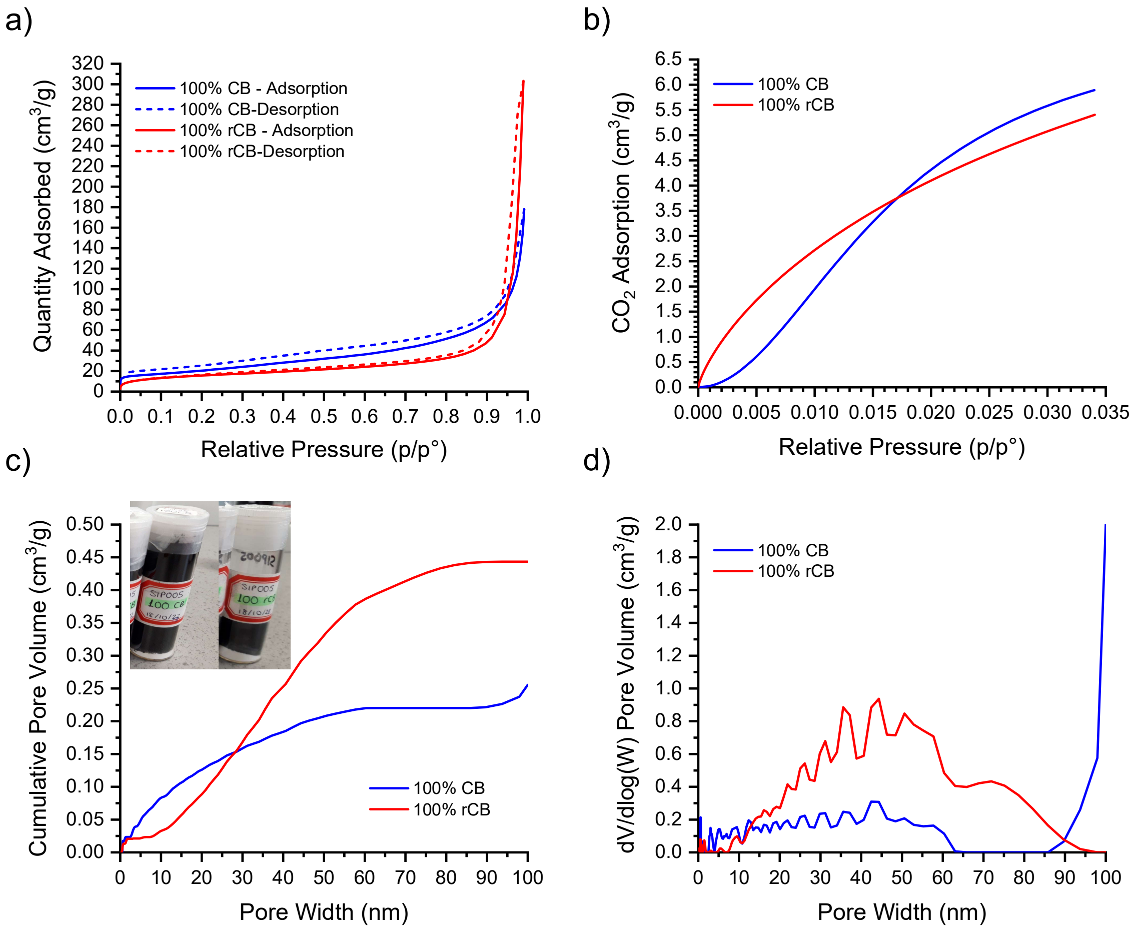

Figure S13. (a) N_2 adsorption/desorption curves, (b) CO_2 adsorption curves, (c) cumulative pore volume and (d) pore volume density as a function of pore width of the CB and rCB carbon black powder samples.

Sample	BET SA (m^2 g^{-1})	V_{ultra} (cm^3 g^{-1})	V_{micro} (cm^3 g^{-1})	V_{meso} (cm^3 g^{-1})	V_{tot} (cm^3 g^{-1})	D (nm)
CB	75	0.018	0.023	0.18	0.26	13.6
rCB	57	0.012	0.021	0.31	0.44	30.9

Table S3. BET specific surface area, volume of ultra micropores (<0.7 nm), volume of micropores (<2.0 nm), volume of mesopores (2-50.0 nm), total volume of pores up to 100 nm, and average pore diameter of the CB and rCB carbon powder samples.

Section 6. Supplementary microscopy and sheet resistance information

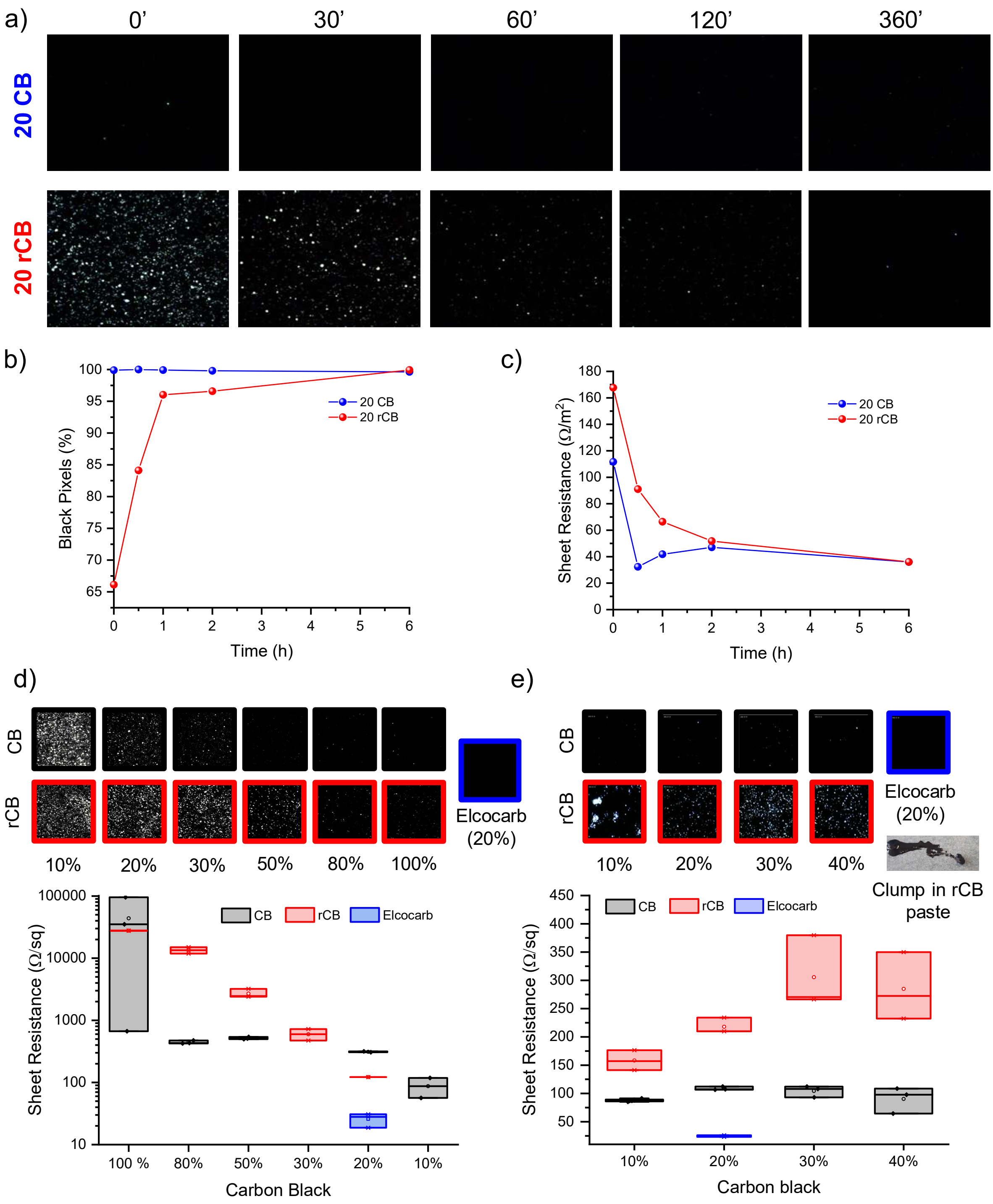

Figure S14. (a) Optical microscope images of films fabricated from different types of carbon paste (20 CB and 20 rCB) that were subjected to increasing sonication times in minutes from 0 to 360. Percentage

of black pixels measured from optical microscope images (b) and sheet resistance (c) of mesoporous carbon films fabricated via doctor blading carbon pastes that were subjected to sonication for different durations. Optical microscope images and sheet resistance values of mesoporous carbon films fabricated from different ratios of carbon black to graphite pastes, made with 15 ml of terpineol (d) and with 8 ml (e).

The sheet resistance of films made from 100% CB, 100% rCB, and lower carbon black percentages was evaluated in Figure S14 d. Overall, the rCB samples exhibited lower conductivity than CB, which aligns with their composition. Conductivity was further hindered by clumping in the paste, leading to pinholes in the films and contributing to high sheet resistance values. These issues were mitigated somewhat by reducing terpineol volume (Figure S14 e) and using sonication to improve carbon black dispersion.

Section 7. Supplementary TRPL information

Equation for fit of the time resolved photoluminescence data:

$$f(t) = A + B_1 e^{-t/\tau_1} + B_2 e^{-t/\tau_2}$$

$$\tau_{Ave} = \frac{B_1 \cdot \tau_1 + B_2 \cdot \tau_2}{B_1 + B_2}$$

τ_{Av} = Amplitude weighted lifetime

	Perovskite	*100% rCB + Perovskite*	*100% CB + Perovskite*	*Elcocarb*	*20% rCB + Perovskite*	*20% CB + Perovskite*
A	0.0064 ± 0.0002	0.0024 ± 0.0001	0.0134 ± 0.0002	0.0113 ± 0.0003	0.0046 ± 0.0002	0.0208± 0.0003
τ_1	39.9 ± 0.5	57.9 ± 0.6	15.0 ± 0.2	37.0 ± 0.5	29.4 ± 0.4	22.2 ± 0.4
τ_2	445 ± 5	380 ± 3	455 ± 8	733 ± 10	344 ± 4	766 ± 10
B_1	7.69 ± 0.27	3.32 ± 0.06	648 ± 66	9.57 ± 0.40	19.6 ± 0.9	59.7 ± 5.2
B_2	0.275 ± 0.004	0.412 ± 0.0048	0.168 ± 0.003	0.202 ± 0.002	0.331 ± 0.004	0.201 ± 0.002
τ_{Ave}	53.9	93.4	15.1	51.4	34.7	24.7

Table S4. Time-resolved PL fit values for the bi-exponential decay.

Various models were tested to fit the data, including multi-exponential decay and stretched exponential fits.[1–3] It was not possible to extract meaningful information from models with three or more components or a global fit due to the heterogeneity of the sample. We therefore base our interpretation on a simple bi-exponential decay which has the appropriate balance of adequately describing the data and having a physical significance.

Section 8. Supplementary device fabrication and optimisation information

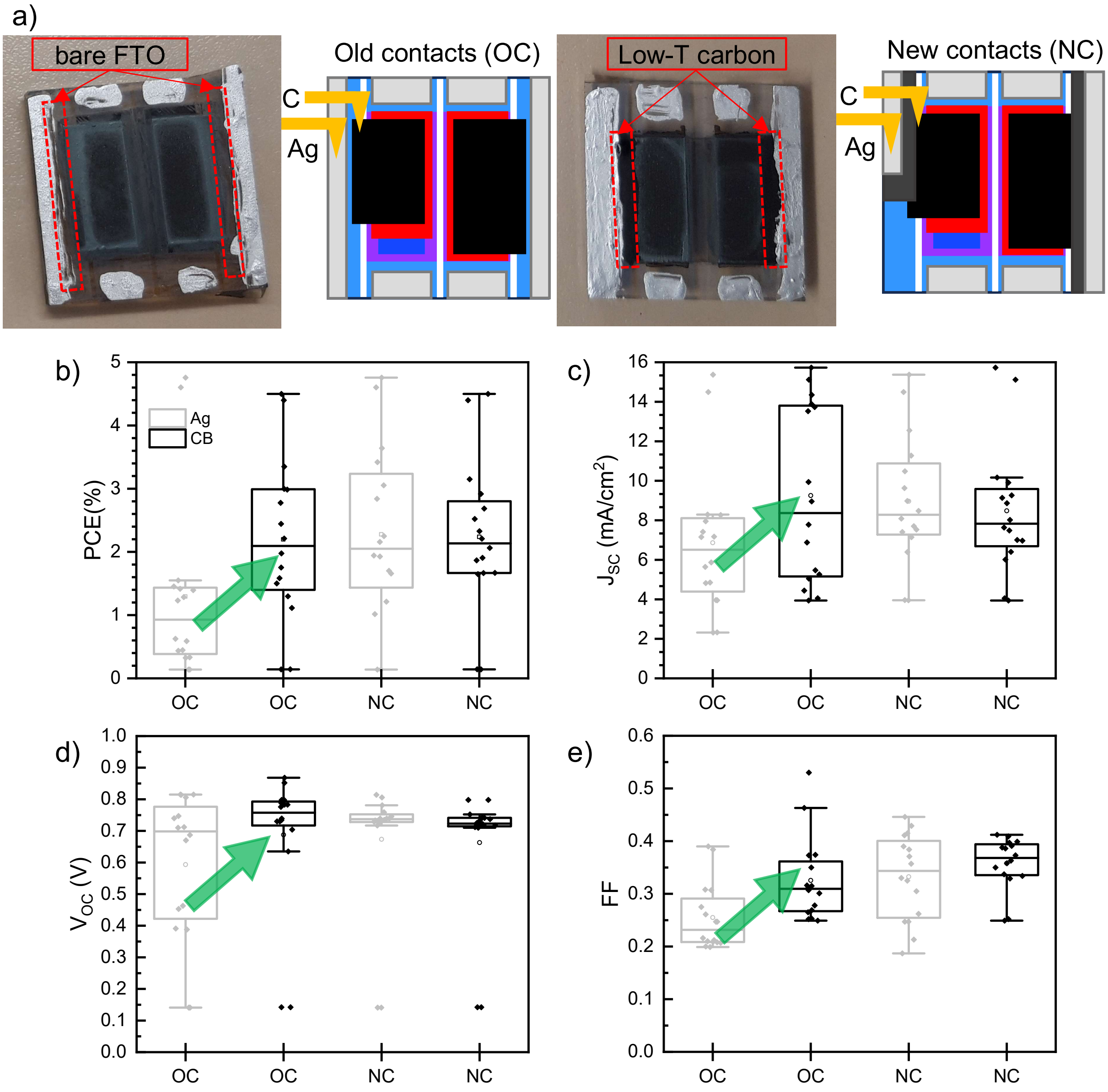

Figure S15. Box plot statistics of the photovoltaic parameters: PCE (b), J_{SC} (c), V_{OC} (d), and FF (e) of solar cells with a layer of low temperature carbon between the mesoporous carbon and the silver contact (NC) and without it (OC). Note the increase in all the cell parameters in the OC samples when measured at the silver versus the mesoporous carbon interfaces as illustrated in (a).

During the device optimisation process (Figures S15 to S21), we note overall low PCEs due to the relatively large active area of the fabricated devices and the limited conductivity of the carbon paste, where the high series resistance dominates. This can be clearly seen in Figure S16 a and Figure S17 a, where the top performing cell from the batch increases in efficiency when the masking area is reduced. The top PCE is therefore reached when the illuminated area is set to 0.0625 cm^2. However, this increased shading significantly reduces the V_{OC} between 0.05 and 0.15 V delivering between 6 - 20% lower PCE than the expected for a true active area of 0.065 cm^2. Some of this large sheet resistance was corrected with the addition of the low temperature carbon layer on the sides, as observed when comparing the JV scan shapes between Figure S16 a and Figure S17 a. Despite this change, the efficiencies in the reference devices are lower compared to other publications, which we can attribute to suboptimal manufacturing conditions arising from a low-cost slot-die coater and high fume hood airflow. Likewise, we acknowledge that the thickness and roughness of the mesoporous layers could be optimized in the future, which is likely to result in lower sheet resistances and better overall performance.

In Figure S17 a, further limitations in the extraction of charge from the mesoporous carbon layer were noticeable in the form of an acute S shape in the reverse scan. Here the charge seems to be extracted favourably until a limit is reached and recombination starts decreasing the available current density. We notice the bump in the JV curve to occur also for the recovered carbon black cells, and to respond to the scan speed (Figure S18), with slower speeds generating a more defined peak than faster speeds. Ion accumulation at the interfaces could be a cause of the limited extraction of the charge since the timescales for ion migration in $MAPbI_3$ have been estimated between milliseconds and seconds at room temperature and under an operational electric field.[4–7] However, charge accumulation at the perovskite/carbon interface can be deemed the dominant cause for the current drop in this case, given the limitations of hole transport in HTL-free carbon devices.[8–11]

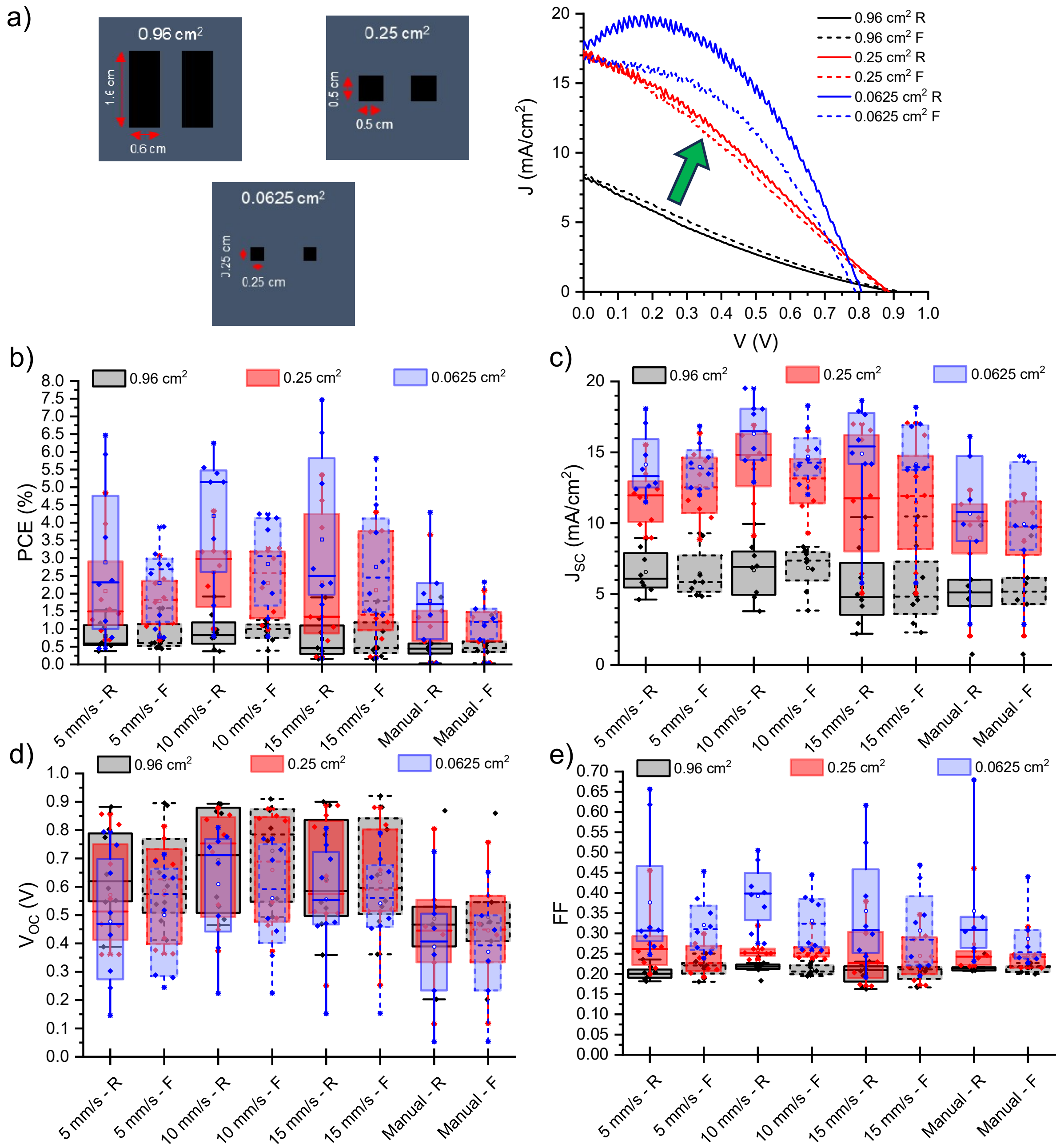

Figure S16. a) Reverse (R) and forward (F) JV scans of the top performing Elcocarb solar cell from the batch measured at different the different mask opening areas (0.96 cm^2, 0.25 cm^2 and 0.0625 cm^2) adjacent to the plot. Box plot statistics of the reverse (R) and forward (F) photovoltaic parameters: PCE (b), J_{SC} (c), V_{OC} (d), and FF (e) of Elcocarb triple mesoscopic stack solar cells slot-die coated at different speeds versus the manual drop-casting of solution.

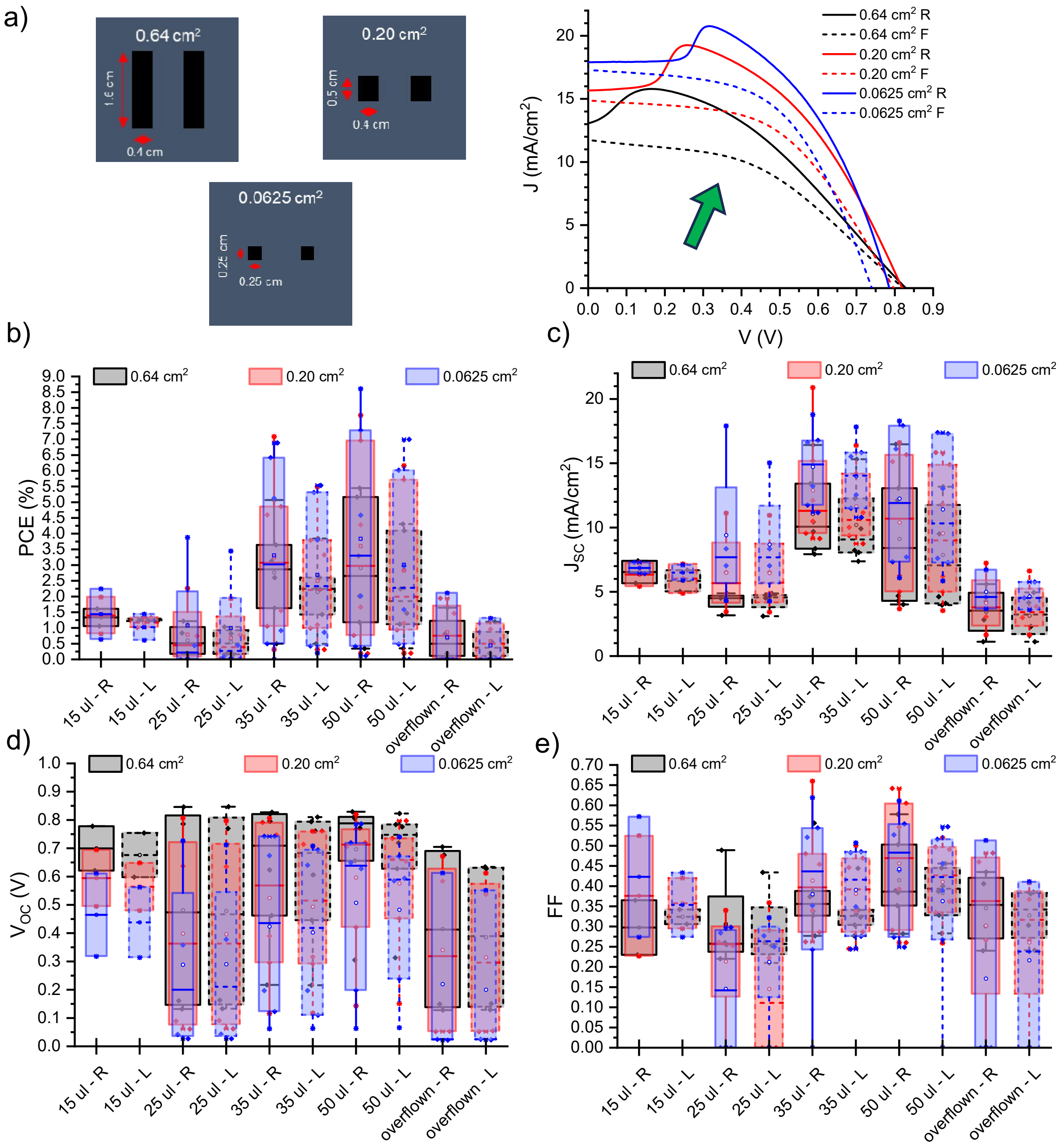

Figure S17. (a) Reverse (R) and forward (F) JV scans of the top performing Elcocarb solar cell from the batch measured at different the different mask opening areas (0.64 cm^2, 0.20 cm^2 and 0.0625 cm^2) adjacent to the plot. Box plot statistics of the reverse (R) and forward (F) photovoltaic parameters: PCE (b), J_{SC} (c), V_{OC} (d), and FF (e) of Elcocarb triple mesoscopic stack solar cells slot-die coated with different volumes of solution. Note the overflown label corresponds to samples deposited with a visibly large but unquantified volume of solution that was expelled accidentally by the slot-die coater when malfunctioning, creating a visibly large capping layer of liquid perovskite on the surface of the samples.

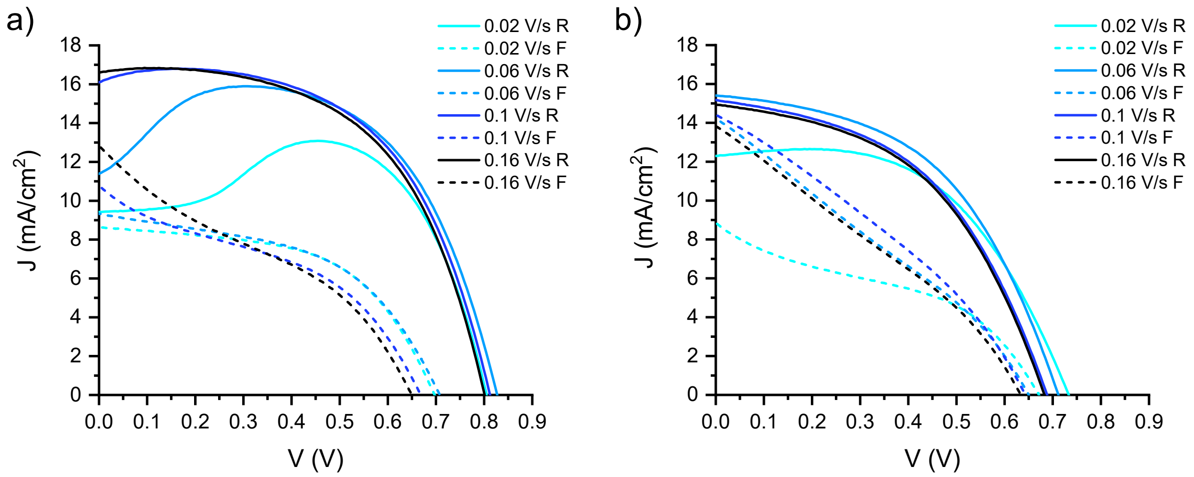

Figure S18. Reverse (R) and forward (F) JV scans performed at different speeds on Elcocarb triple mesoscopic perovskite cells presenting a noticeable (a) and no noticeable (b) bump on the curve at a standard scan speed of 0.06 V s^{-1}.

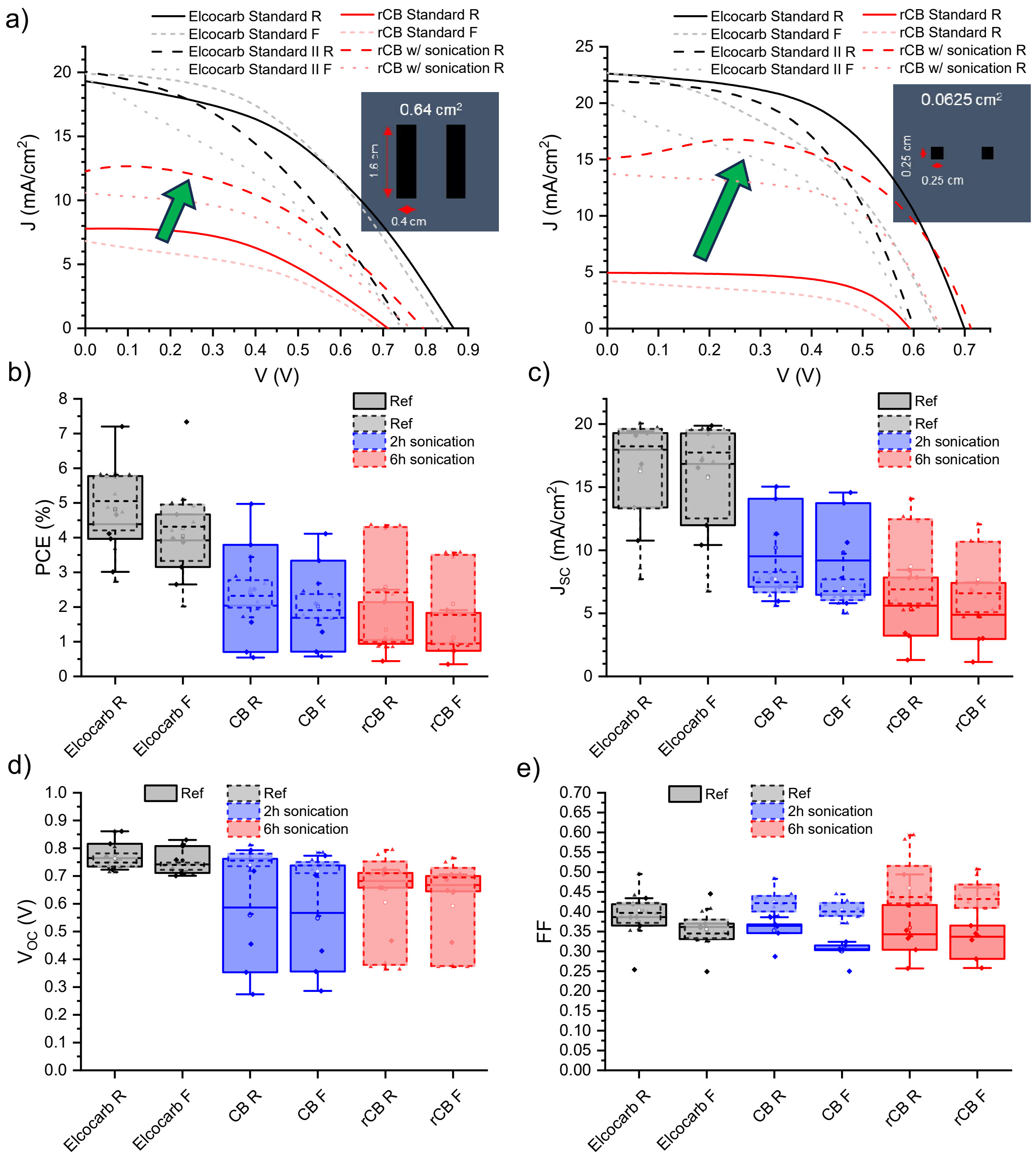

Figure S192. Reverse (R) and forward (F) JV scans of the top performing reference Elcocarb and 20 rCB solar cells from two batches: one where a sonication treatment was performed on the lab made pastes and one without the sonication treatment, measured with an opening mask of 0.64cm^2 (a) and 0.0625 cm^2 (b). Box plot statistics of the reverse (R) and forward (F) photovoltaic parameters: PCE (b), J_{SC} (c), V_{OC} (d), and FF (e) of two batches of Elcocarb, CB and rCB devices. One where the CB and rCB pastes were not sonicated (solid line) and another where the CB and rCb pastes were sonicated for 2h and 6h, respectively (dashed line).

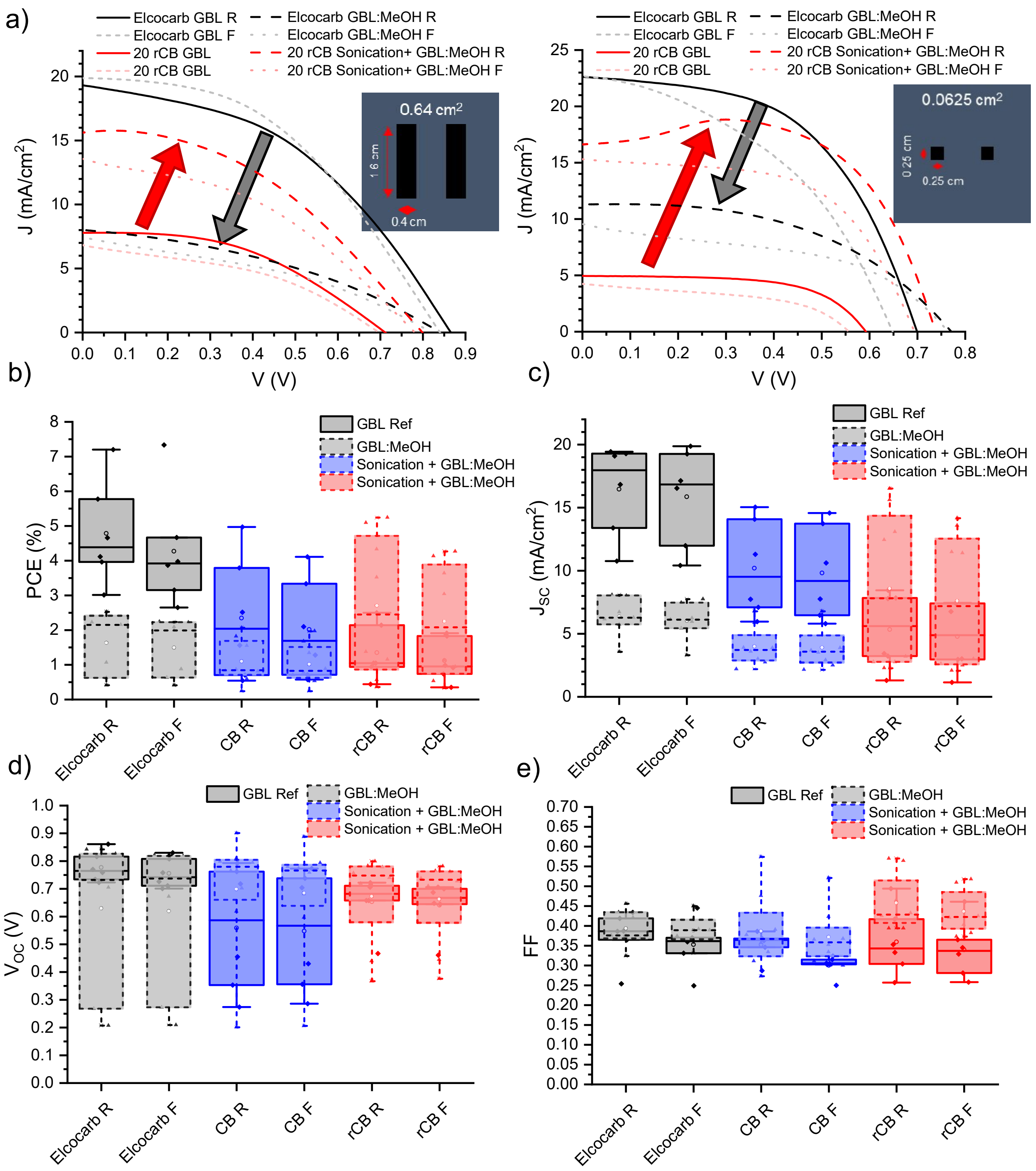

Figure S20. Reverse (R) and forward (F) JV scans of the top performing reference Elcocarb and 20 rCB solar cells from two batches: one where a sonication treatment was performed on the lab made pastes and a mixture of GBL:MeOH was used as a solvent for the perovskite solution, and one without the sonication treatment and the standard GBL solution, measured with an opening mask of 0.64cm^2 (a) and 0.0625 cm^2 (b). Note how the use of the GBL:MeOH solution was not favourable for the commercial Elcocarb sample while the combination of a sonication treatment and the GBL:MeOH solution increased the performance of the rCB sample. Box plot statistics of the reverse (R) and forward (F) photovoltaic

parameters: PCE (b), J_{SC} (c), V_{OC} (d), and FF (e) of two batches of Elcocarb, CB and rCB devices. One where the CB and rCB pastes were not sonicated (solid line) and use the standard perovskite GBL solution and another where the CB and rCB pastes were sonicated for 2h and 6h, respectively and a GBL:MeOH solution was used (dashed line).

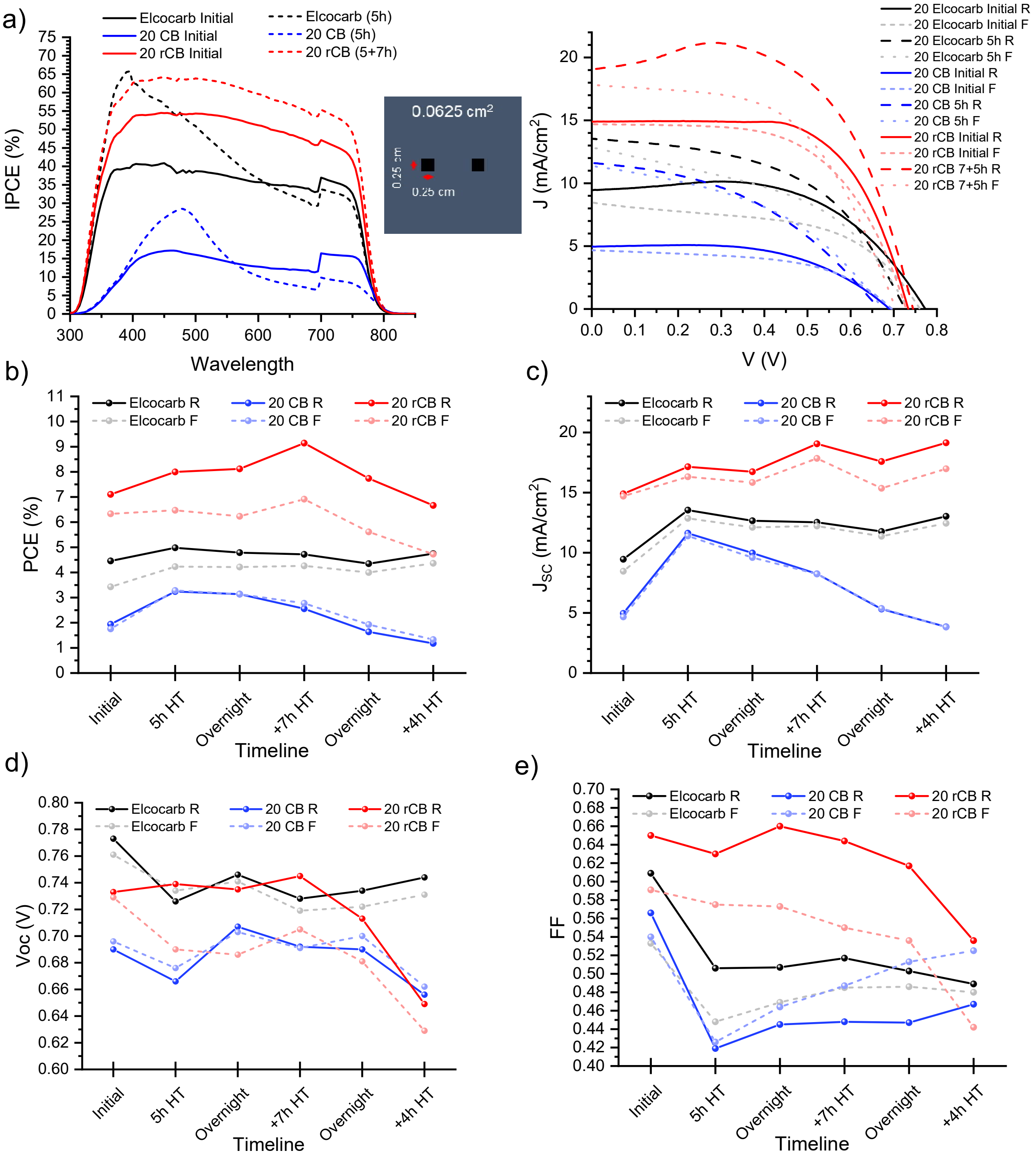

Figure S21. (a) IPCE (left) and reverse (R) and forward (F) JV scans (right) of the top performing Elcocarb, 20% CB and 20% rCB samples before and after being subject to a hydration treatment (40°C,

75%RH) for their respective optimum times, measured with a 0.065 cm^2 mask. Photovoltaic parameters of the reverse (R) and forward (F) scans: PCE (b), J_{SC} (c), V_{OC} (d), and FF (e) of top performing Elcocarb, sonicated 20% CB and sonicated 20%rCB cells slot-die coated with a GBL:MeOH solution over time. The hydration treatment was performed for 5 hours initially (5h HT) and samples were stored overnight in the desiccator (Overnight). The following day samples were subject to 7 hours of hydration treatment (+7 HT), left overnight for a second time in the desiccator (Overnight) and subject to 4 more hours of treatment on the following day (+4 HT).

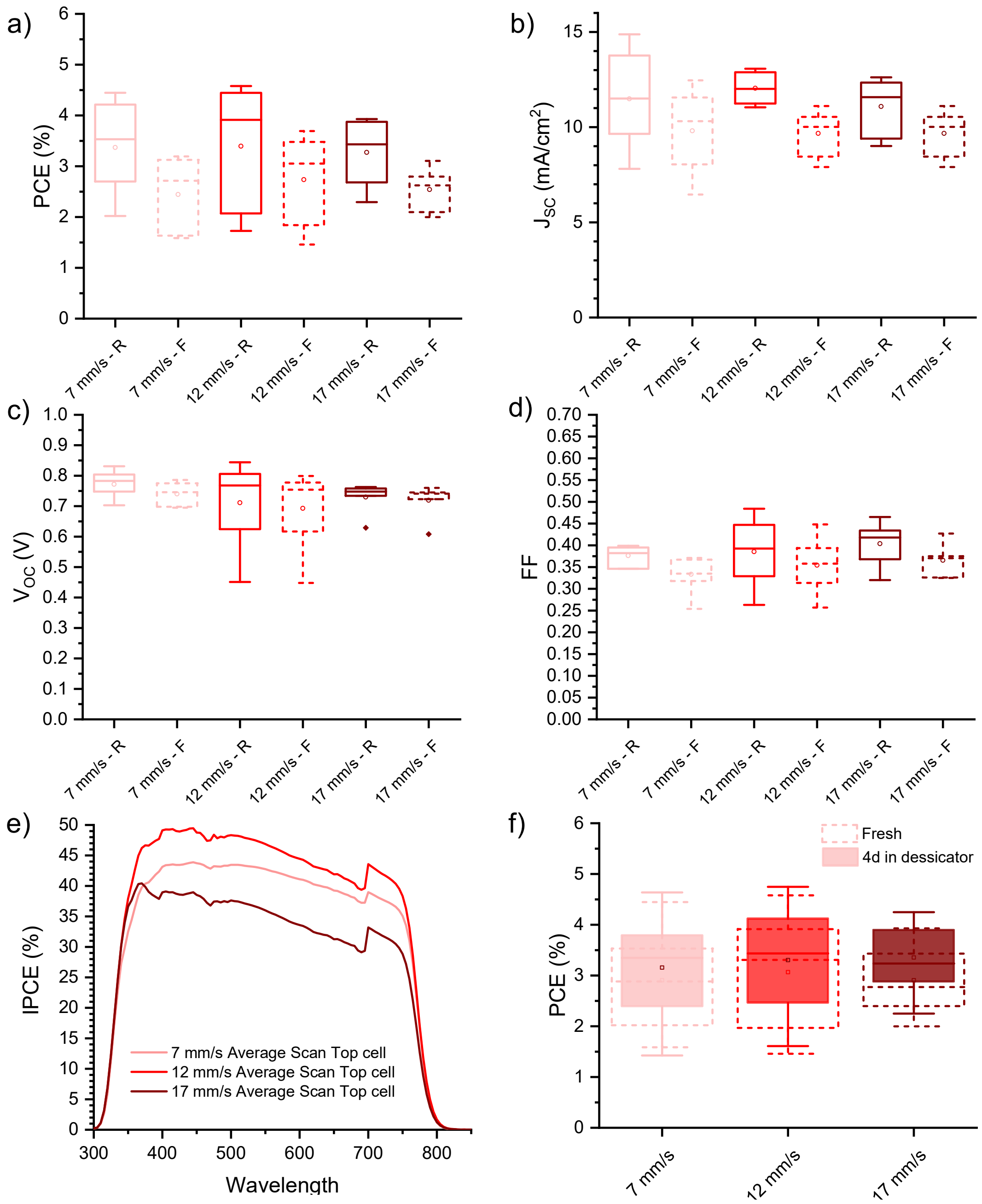

Figure S22. Box plot statistics of the reverse (R) and forward (F) photovoltaic parameters: PCE (a), J_{SC} (b), V_{OC} (c), and FF (d) of 20% rCB solar cells slot-die coated at different speeds. (e) IPCE of the top performing cells per speed of the batch measured with a 0.0625 cm^2 mask. (f) Boxplot statistics of the PCE of the batch after fabrication (Fresh) versus after 4 days stored in the desiccator.

Section 9. Supplementary Outdoor Test information

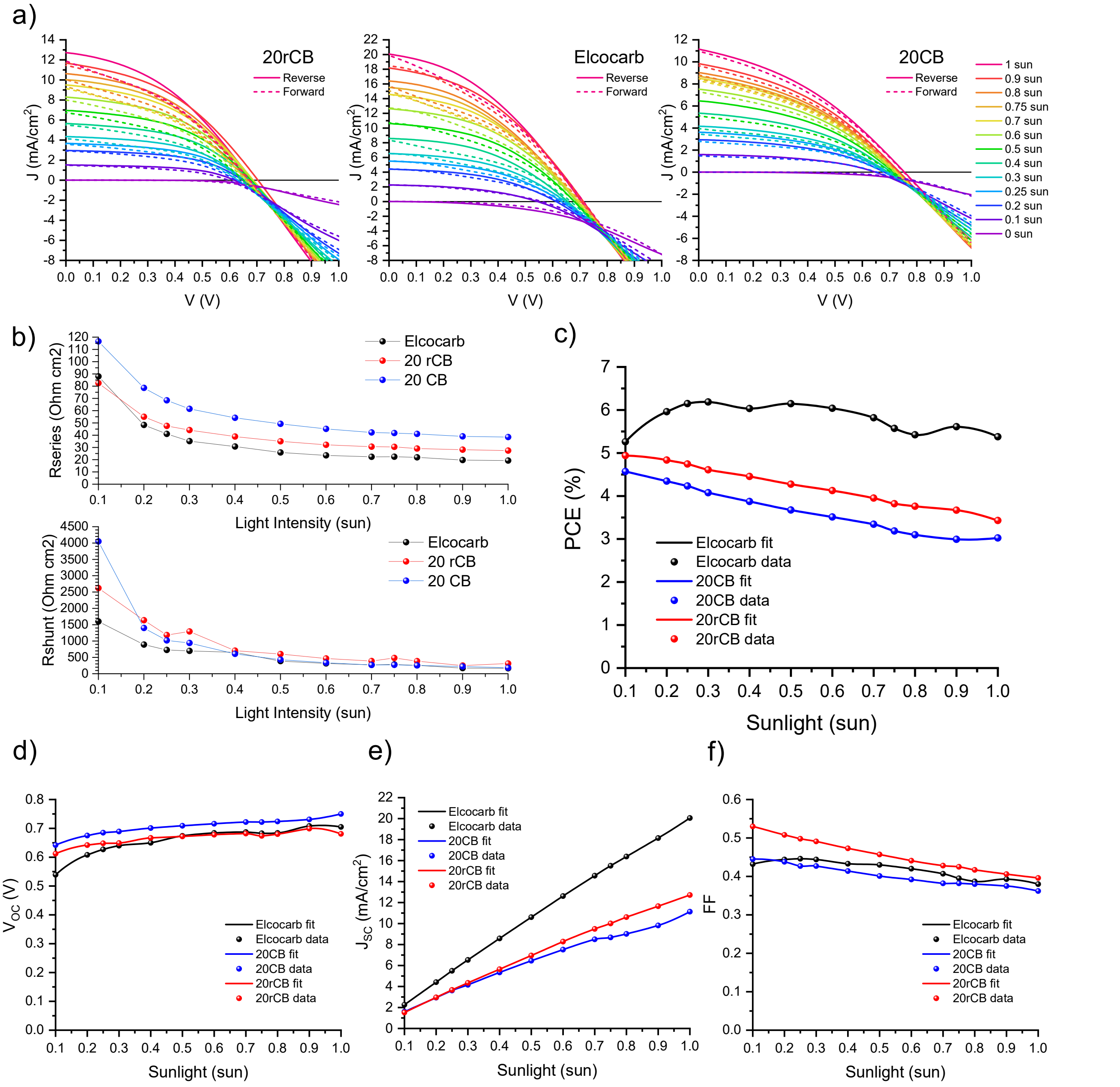

Figure S23. (a) Reverse (solid line) and forward (dashed line) JV scans of 20% rCB, Elcocarb and 20% CB cells under 0, 0.1, 0.2, 0.25, 0.3, 0.4, 0.5, 0.6, 0.7, 0.75, 0.8, 0.9, and 1 sun light intensities before outdoor deployment. (b) Series and shunt resistance of the JV scans plotted in (a) as a function of light

intensity. The reverse photovoltaic parameters of cells in (a) plotted as a function of light intensity are represented in (c)-PCE, (d) $-J_{SC}$, (e) $-V_{OC}$, and (f) – FF.

The JV curves of outdoor-deployed solar cells (Figure S23 a), measured with a 0.64 cm^2 mask area at various light intensities and in the dark, reveal that series resistance dominates their performance. This is reflected in the steeper bending of the JV curves near open-circuit voltage at lower light intensities. These effects are showcased more clearly in Figure S23 b, where both resistances are plotted against light intensity. High series resistance effects can be normally attributed in triple mesoscopic stacks to the engineering of the mesoporous layers. Relatively rough surfaces, defects at the mesoporous interfaces, large thicknesses of some layers as well as their pore size contribute to higher series resistance. We understand that further optimization should be done in the future on those layers to achieve less sheet resistance and therefore improve the performance of the devices.

On the other hand, shunt resistance shows a diminishing effect at lower light intensities, with dark leakage current values of 0.01061 mA/cm^2 in the Elcocarb sample, 0.00805 mA/cm^2 in the 20 rCB sample and 0.00852 mA/cm^2 in the 20 CB sample.

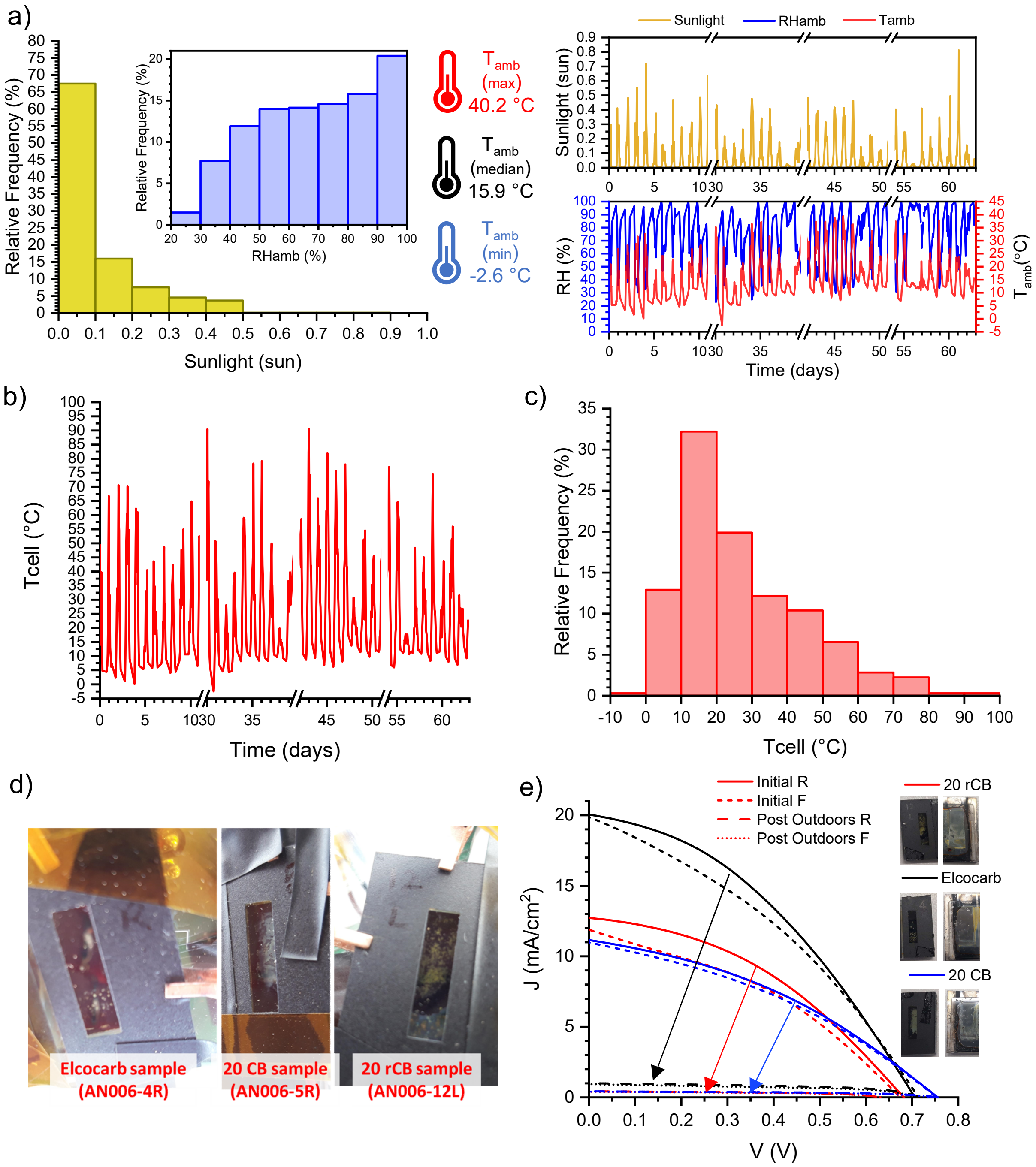

Figure S24. (a) Outdoor weather conditions measured by the sensors including a histogram of light intensity and relative humidity, maximum, median, and minimum ambient temperatures registered in the box, and variations in light intensity, ambient temperature and humidity during the measurement. (b) Temperature recorded on the surface of the 20% rCB cell over time during the outdoor test. (c) Histogram representing the relative frequency of temperatures at the surface of the 20% rCB cell recorded over the outdoor deployment. (d) Pictures of the Elcocarb, 20%CB and 20% rCB samples after one month of

outdoor deployment. (e) JV scans before and after outdoor testing of the Elcocarb, 20% CB and 20% rCB devices with images.

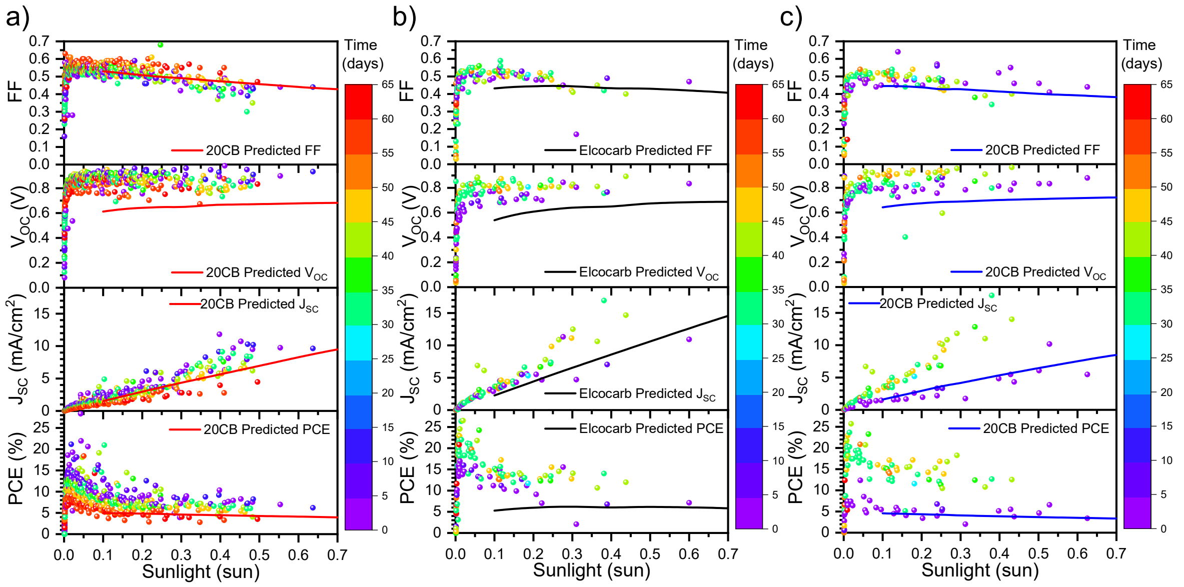

Figure S25. Reverse photovoltaic parameters plotted as a function of light intensity of the 20% rCB (a), Elcocarb (b) and 20% CB recorded during the outdoor deployment. The solid line represents the Akima interpolation fits acquired during the calibration before the outdoor deployment. The colormap groups the PV parameters in a timeframe starting at the first day of outdoor measurement (day 0).

REFERENCES

(1) Péan, E. V.; Dimitrov, S.; De Castro, C. S.; Davies, M. L. Interpreting Time-Resolved Photoluminescence of Perovskite Materials. *Physical Chemistry Chemical Physics* 2020, *22* (48), 28345–28358. https://doi.org/10.1039/D0CP04950F.

(2) Chen, X.; Kamat, P. V.; Janáky, C.; Samu, G. F. Charge Transfer Kinetics in Halide Perovskites: On the Constraints of Time-Resolved Spectroscopy Measurements. *ACS Energy Lett* 2024, *9* (6), 3187–3203. https://doi.org/10.1021/acsenergylett.4c00736.

(3) Taddei, M.; Jariwala, S.; Westbrook, R. J. E.; Gallagher, S.; Weaver, A. C.; Pothoof, J.; Ziffer, M. E.; Snaith, H. J.; Ginger, D. S. Interpreting Halide Perovskite Semiconductor Photoluminescence Kinetics. *ACS Energy Lett* 2024, *9* (6), 2508–2516. https://doi.org/10.1021/acsenergylett.4c00614.

(4) Jacobs, D. A.; Wu, Y.; Shen, H.; Barugkin, C.; Beck, F. J.; White, T. P.; Weber, K.; Catchpole, K. R. Hysteresis Phenomena in Perovskite Solar Cells: The Many and Varied Effects of Ionic Accumulation. *Physical Chemistry Chemical Physics* 2017, *19* (4), 3094–3103. https://doi.org/10.1039/C6CP06989D.

(5) Li, C.; Guerrero, A.; Zhong, Y.; Huettner, S. Origins and Mechanisms of Hysteresis in Organometal Halide Perovskites. *Journal of Physics: Condensed Matter* 2017, *29* (19), 193001. https://doi.org/10.1088/1361-648X/aa626d.

(6) Eames, C.; Frost, J. M.; Barnes, P. R. F.; O'Regan, B. C.; Walsh, A.; Islam, M. S. Ionic Transport in Hybrid Lead Iodide Perovskite Solar Cells. *Nat Commun* 2015, *6* (1), 7497. https://doi.org/10.1038/ncomms8497.

(7) Richardson, G.; O'Kane, S. E. J.; Niemann, R. G.; Peltola, T. A.; Foster, J. M.; Cameron, P. J.; Walker, A. B. Can Slow-Moving Ions Explain Hysteresis in the Current–Voltage Curves of Perovskite Solar Cells? *Energy Environ Sci* 2016, *9* (4), 1476–1485. https://doi.org/10.1039/C5EE02740C.

(8) De Moor, G.; Charvin, N.; Farha, C.; Meyer, T.; Perrin, L.; Planes, E.; Flandin, L. Understanding the Anomalous *J–V* Curves in Carbon-Based Perovskite Solar Cells as a Structural Transition Induced by Ion Diffusion. *Solar RRL* 2024, *8* (8). https://doi.org/10.1002/solr.202300998.

(9) Li, S.; Li, Y.; Sun, X.; Li, Y.; Deng, F.; Tao, X. Hole Transport Layer-Free Carbon-Based Perovskite Solar Cells with High-Efficiency up to 17.49% in Air: From-Bottom-to-Top Perovskite Interface Modification. *Chemical Engineering Journal* 2023, *455*, 140727. https://doi.org/10.1016/j.cej.2022.140727.

(10) Zhang, T.; Liu, C.; Li, Z.; Zhao, B.; Bai, Y.; Li, X.; Liu, W.; Chen, Y.; Liu, Z.; Li, X. Improved Perovskite/Carbon Interface through Hot-Pressing: A Case Study for CsPbBr3 -Based Perovskite Solar Cells. *ACS Omega* 2022, *7* (20), 16877–16883. https://doi.org/10.1021/acsomega.1c06108.

(11) Liu, H.; Geng, C.; Wei, P.; Chen, H.; Zheng, S.; Wang, H.; Xie, Y. Improving the Performance and Stability of Large-Area Carbon-Based Perovskite Solar Cells Using N, O Co-Doped Biomass Porous Carbon. *J Alloys Compd* 2022, *912*, 165123. https://doi.org/10.1016/j.jallcom.2022.165123.
